# Wettability and Bactericidal Properties of Bioinspired ZnO Nanopillar Surfaces

**DOI:** 10.1021/acs.langmuir.3c03537

**Published:** 2024-03-27

**Authors:** Jitao Zhang, Georgia Williams, Thanaphun Jitniyom, Navdeep Sangeet Singh, Alexander Saal, Lily Riordan, Madeline Berrow, James Churm, Manuel Banzhaf, Felicity de Cogan, Nan Gao

**Affiliations:** † School of Engineering, University of Birmingham, Edgbaston ,Birmingham B15 2TT, United Kingdom; ‡ School of Biosciences, University of Birmingham, Edgbaston ,Birmingham B15 2TT, United Kingdom; § School of Pharmacy, University of Nottingham, University Park, Nottingham NG7 2RD, United Kingdom

## Abstract

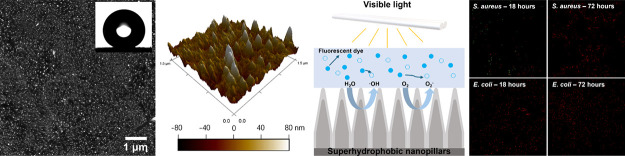

Nanomaterials of zinc oxide (ZnO) exhibit antibacterial activities under ambient illumination that result in cell membrane permeability and disorganization, representing an important opportunity for health-related applications. However, the development of antibiofouling surfaces incorporating ZnO nanomaterials has remained limited. In this work, we fabricate superhydrophobic surfaces based on ZnO nanopillars. Water droplets on these superhydrophobic surfaces exhibit small contact angle hysteresis (within 2–3°) and a minimal tilting angle of 1°. Further, falling droplets bounce off when impacting the superhydrophobic ZnO surfaces with a range of Weber numbers (8–46), demonstrating that the surface facilitates a robust Cassie–Baxter wetting state. In addition, the antibiofouling efficacy of the surfaces has been established against model pathogenic Gram-positive bacteria *Staphylococcus aureus* (*S. aureus*) and Gram-negative bacteria *Escherichia coli* (*E. coli*). No viable colonies of *E. coli* were recoverable on the superhydrophobic surfaces of ZnO nanopillars incubated with cultured bacterial solutions for 18 h. Further, our tests demonstrate a substantial reduction in the quantity of *S. aureus* that attached to the superhydrophobic ZnO nanopillars. Thus, the superhydrophobic ZnO surfaces offer a viable design of antibiofouling materials that do not require additional UV illumination or antimicrobial agents.

## Introduction

The formation of biofilms on the surface of medical devices and implants is a major cause of nosocomial or healthcare-associated infections.^1−5^ The spreading and adhesion of bacteria are often more severe in the presence of liquid, for example, in the forms of slug flow and intermittent droplets.^6−9^ It is therefore necessary to design surfaces that are not only liquid repellent but also resistant to biological contamination in order to prevent initial bacterial attachment. In recent years, there has been a lot of research toward the use of superhydrophobic surfaces to reduce bacterial adhesion and, thus, biofilm formation.^10−13^ Superhydrophobic surfaces often rely on low-surface-energy coatings on nanostructures with a particular roughness. This allows a droplet to exhibit the so-called Cassie–Baxter wetting state on the surface with large contact angles greater than 150°.^14^ More specifically, the key feature of the Cassie–Baxter state is that the droplet is partially supported by the surface nanostructure and partially suspended on air pockets entrapped underneath the droplet.^15−18^

The presence of air pockets minimizes the effective contact area between the liquid (e.g., bacteria-containing droplets) and the surface, thereby reducing interfacial adhesion. By contrast, recent studies have even demonstrated that the absence of air pockets greatly promotes bacterial adhesion.^19−22^ Further, natural superhydrophobic surfaces such as cicada wings that facilitate the Cassie–Baxter wetting state have been shown to feature bactericidal properties, which are attributed to their rigid surface nanopillars resulting in direct bacterial membrane puncture.^23,24^ It is thus desirable to make a surface intrinsically superhydrophobic and bactericidal, in order to achieve long-term antibiofouling effect. One of the approaches commonly used to make superhydrophobic surfaces is to coat a structured substrate with polydimethylsiloxane (PDMS), which is hydrophobic with static contact angles on the order of 100°.^25,26^ PDMS, due to its flexibility, transparency, and biocompatibility, has been applied in a wide range of industrial and scientific applications, especially in biomedical devices, such as catheters and microfluidic devices.^27−29^ However, PDMS is generally considered to be biologically inert, despite its inherent hydrophobicity. This means that PDMS does not interact with or directly affect microorganisms, and therefore, it does not have inherent antimicrobial properties of its own. As such, PDMS coatings can become contaminated and retain bacteria that are able to multiply and form biofilms in the absence of appropriate cleaning and sterilization protocols.^30^

By contrast, metal-oxide photocatalysts, such as TiO_2_ and ZnO, have demonstrated antimicrobial properties, which are attributed to the reactive free radicals generated under illumination.^31−36^ Specifically, their photocatalytic activities can either lead to cell membrane disorganization or induce oxidative stress in bacterial cells. Of particular interest is ZnO, which can exhibit photocatalytic activities under ambient illumination.^31−33^ Yet, ZnO is intrinsically hydrophilic, which is undesired for applications that require low interfacial adhesion. In this work, we report the development of superhydrophobic surfaces based on PDMS-coated ZnO nanopillars that exhibit promising antibiofouling properties. Droplets of water on these superhydrophobic surfaces exhibit a large advancing contact angle of around 154° and a receding contact angle of around 152°, enabling a low tilt angle of 1°. Furthermore, the experimental results show that the superhydrophobic surfaces of PDMS-coated ZnO nanopillars can effectively kill Gram-negative bacterium *Escherichia coli* (*E. coli*) and inhibit the Gram-positive bacterium *Staphylococcus aureus* (*S. aureus*). The results reported here generate insight into the combination of superhydrophobicity and bactericidal activity, which could facilitate a platform for the design of antibiofouling materials.

## Experimental Section

### Materials

Pure zinc foils (99.9%, 0.62 mm in thickness) were purchased from Fisher Scientific (UK) as the substrate for the fabrication of ZnO nanopillars. Sodium hydroxide (NaOH) pellets and zinc nitrate hexahydrate pellets (Zn(NO_3_)_2_·6H_2_O) were purchased from Sigma-Aldrich (UK). Polydimethylsiloxane (PDMS), SYLGARD 184 Silicone elastomer curing agent, and ethyl acetate (Sigma-Aldrich, UK) were used to coat the ZnO nanopillars. Ethanol, acetone, and deionized (DI) water were used to clean the bare substrates. *S. aureus* and *E. coli* were used in the bactericidal efficacy testing. The LIVE/DEAD BacLight bacterial viability kit was purchased from Fisher Scientific (UK).

### Devices

#### Goniometer

Contact angle (CA) measurements were made with a DataPhysics OCA15EC goniometer. The static CAs were measured by dosing a water droplet of up to 12 μL on bare zinc, ZnO nanopillars, or PDMS-coated nanopillar surfaces. For the dynamic CAs (advancing and receding contact angles), the volume of the droplet was increased or decreased at a dosing rate of 1 μL/s each time. The image and video results were captured by the 6.5-fold zoom lens of the goniometer itself.

#### X-ray Diffractometer

X-ray diffraction (XRD) data was collected using a Proto AXRD Benchtop diffractometer (Proto, USA) operated at 30 kV and 20 mA with a Cu Kα source (λ = 1.54251 Å). The samples were analyzed over the 2θ range of 20–80° using 0.0149° increments.

#### Fourier Transform Infrared Spectrometer

Fourier transform infrared (FTIR) results were obtained using a Nicolet iS50 FTIR spectrometer. All spectra were recorded in the range of 4000 to 400 cm^–1^, with a spectral resolution of 4 cm^–1^ in both absorbance and reflectance mode with the ATR module. Measurements were taken with 256 coadded scans.

#### Microplate Reader

Photocatalytic performance was measured using a BMG Labtech CLARIOstar Plus plate reader in fluorescence intensity mode to quantify the degradation rate of fluorescent dye CF633. An excitation wavelength of 625 ± 10 nm and an emission wavelength of 650 ± 20 nm were set for the fluorescence intensity mode. Thirty microliters of 0.01 mg/mL fluorescent dye was placed on different surfaces. Degradation was monitored indoors under ordinary lighting conditions with a light intensity of 400 lx, and the distance between the light source and the sample was 2.5 m. The experiment was repeated three times under the same lighting condition, with readings taken every 1 h.

#### Scanning Electron Microscopy (SEM)

SEM images were taken using Zeiss EVO 10 Materials SEM and Thermo Scientific Apreo 2S SEM at an acceleration voltage of 5.0–15.0 kV. PDMS-coated samples were coated with 8 nm of Au by sputter deposition before analysis. Bare zinc and ZnO nanopillar samples offered good conductivity but were also coated with gold layers to ensure consistency of the observation parameters.

#### Atomic Force Microscopy (AFM)

AFM studies were carried out using a Cypher S AFM instrument from Oxford Instruments. The AFM images were taken using the air contact mode (tapping mode), with a scan size of 3 × 3 or 1 × 1 μm^2^ at a scan rate of 1.0 Hz.

#### High-Speed Videos

Water droplet impact behaviors were captured using a high-speed camera (FASTCAM Mini UX100, Photron) at a frame rate of 1000 fps.

#### Confocal Microscopy

Fluorescent images of LIVE/DEAD bacterial populations were captured using the Leica TCS SP8 confocal microscope (with a 40×/1.40 oil objective). Immersion oil (Immersol 518 F) was utilized to cover the sample surfaces. The excitation wavelength was at 488nm for the LIVE green signal SYTO 9 and 561 nm for the DEAD red signal propidium iodide.

### Fabrication and Modification of ZnO Nanopillars

The zinc foils were cut into small substrates with the dimensions of 1 × 1 cm. Since zinc foils are very susceptible to oxidation, they were first polished with fine sandpaper to remove organic residues and natural oxide layers before washing with copious amounts of DI water. The zinc substrates were then ultrasonically degreased in ethanol, acetone, and DI water for 5 min each. The surfaces were afterward dried with nitrogen (N_2_) flow and stored under desiccation in a sealed container or vacuum desiccator. Subsequently, a two-step fabrication process was used to fabricate superhydrophobic ZnO nanopillar structures on zinc foil surfaces. A hydrothermal synthesis method was used as the first step to grow ZnO nanopillars. Typically, 35 mL of 0.43 mol/L zinc nitrate hexahydrate (Zn(NO_3_)_2_·6H_2_O) was poured into 35 mL of 3.43 mol/L sodium hydroxide (NaOH) to form an alkaline zincate solution. The cleaned zinc substrate was then dipped into the solution and placed on a stirring hot plate with the beaker sealed at 100 °C for 45 min. During this process, a concentration gradient of zincate ions was created near the zinc substrate, eventually forming ZnO nanopillars.^37^ After 45 min, the zinc substrate was removed from the solution and dried using nitrogen flow after rinsing with DI water. Then, the wettability of the nanopillars was modified by coating the surfaces with a thin PDMS layer. Typically, the PDMS solution was prepared by mixing 1 mL of PDMS, 0.1 mL of silicone elastomer curing agent, and 100 mL of ethyl acetate. It was stirred at 250–280 rpm in a beaker at room temperature. After the solution preparation, the surfaces of ZnO nanopillars were drop-casted with 5 μL of well-stirred PDMS solution for a thin layer of coating and then placed in an oven at 80 °C for 6 h to cure.

### Bacterial Culture Preparation

Both *E. coli* ATCC25922 and *S. aureus* ATCC6528 were cultured in 5 mL of lysogeny broth (LB) overnight at 37 °C for ≈18 h with shaking at 180 rpm. The culture was then adjusted to ≈1 × 10^9^ (colony-forming units) CFU mL^–1^.

### Agar Plate Preparation

LB Agar was prepared following the manufacturer’s instructions and autoclaved for sterility. After removal from the autoclave, a brief cooling period of approximately 5 min was allowed, followed by gentle shaking to ensure homogeneity. The agar was then poured into plates near a Bunsen flame, ensuring coverage of the bottom of the plate with the agar. A minimum of 12 plates (with three plates for each type of sample, including control, bare zinc, ZnO nanopillars, and PDMS-coated nanopillars) were prepared for the experiment. Each agar plate was sectioned into eight segments labeled from A to H, as shown in Figure S1a.

### Antibiofouling Efficacy Testing

All sample surfaces were incubated with cultured bacterial solutions for 18 and 72 h. The surfaces were inoculated with the bacterial suspensions in a 3 × 3 grid of 1 μL aliquots of bacterial culture across the sample surfaces, as shown in Figure S1b. The PDMS-coated ZnO nanopillar surfaces were an exception because of their superhydrophobicity, which allowed small droplets to roll off easily. Instead, a single 9 μL droplet was carefully pipetted onto the surface of PDMS-coated ZnO nanopillars. After the incubation, the samples were transferred into 15 mL centrifuge tubes containing 10 mL of Dey-Engley neutralizing broth, each accompanied by 7–10 sterile zirconium beads. Subsequently, the tubes were vortex-mixed for 1 min. Serial dilution of 1:3 (eight times) in sterile phosphate-buffered saline (PBS) within a 96-well plate was then carried out using the neutralizing broth suspension. Each row of broth corresponded to the previously sectioned agar plate.

Subsequently, three 10 μL droplets from each row of the PBS solutions that had been serially diluted in the 96-well plate were spotted in their designated sections on the LB agar plates, as shown in Figure S1c. Three sets of repeats for each surface were prepared. The plates were then allowed to dry thoroughly around a Bunsen burner. Once all plates had dried, they were placed in a static incubator at 37 °C for 16–18 h. The plates were removed from the incubator the following day, and the survival of the bacteria was assessed by counting colony-forming units (CFU), where colony numbers were multiplied by the relevant dilution factor.^38^ The limit of detection for the assay was set to 1 × 10^3^ CFU.

### Samples Fixation for Scanning Electron Microscopy (SEM)

Bacterial suspensions were incubated on the surfaces as per the test procedure “antibiofouling efficacy testing” described previously. After 16–18 h of incubation, samples were fixed in 4% paraformaldehyde for 1 h, followed by dehydration in a series of ethanol solutions. These solutions consisted of 5 mL of mixtures of PBS/ethanol, with ethanol content increasing incrementally from 10 to 100% at the concentrations of 10, 30, 50, 70, 90, and 100%. Samples were incubated in each ethanol solution for 10 min and twice in the 100% ethanol solution. Following the treatment with these solutions, the samples were subjected to a 10 min incubation in hexamethyldisilazane (HMDS) solution. They were then placed in a fume hood to allow sufficient time for the evaporation and drying. Following this time, the samples were further prepared for observation by using SEM.

### Confocal Microscopy

Preparation for confocal microscopy was performed following bacterial incubation on the surfaces described in the test procedure “antibiofouling efficacy testing”. After the incubation period, each surface was gently washed with PBS (up to 40 μL) to ensure removal of any residual media. The surfaces were then covered with glass coverslips before using the confocal laser scanning microscope. One to two drops of immersion oil (Immersol 518 F) were added to the top of the glass coverslip for the imaging process to increase the resolution of the microscope.

### Multicycle Surface Tests

A series of multicycle surface tests were performed across 3 × 18 or 5 × 18 h of incubation periods. The “antibiofouling efficacy testing” procedure was then followed for each multicycle test. Each test was subjected to a set of three repeats per surface type. After each test cycle, the surfaces were gently washed by using fresh sterile water. The surfaces were then left to dry thoroughly at room temperature with good ventilation for 1 h before being used for the next set of tests.

### Cytotoxicity Tests

Murine 3T3 fibroblast cells were cultured in Dulbecco’s Modified Eagle Medium (DMEM) supplemented with 10% newborn calf serum, 4 mM GlutaMAX, 100 IU/mL penicillin, 100 μg/mL streptomycin, and 20 mM HEPES (4-(2-hydroxyethyl)-1-piperazineethanesulfonic acid). The revived cell solutions were then seeded in Corning T75 cell culture flasks and incubated for 48 h at 37 °C with 5% CO_2_. Subsequently, cell attachment and growth were monitored under a phase-contrast microscope, and fresh culture medium was replenished every 2–3 days. When a confluent monolayer was observed, cells were passaged using trypsin-EDTA (0.25%), distributed into new T75 flasks, and confirmed the detachment of cells under the microscope. In this process, the number of viable cells could be determined by measuring a 20-μL aliquot of cell suspension mixed with an equal volume of Gibco Trypan blue stain (0.4%) using the Invitrogen Countess automated cell counter.

3T3 cells were then seeded onto various surfaces. After 15 min of incubation at 37 °C with 5% CO_2_ to facilitate cell adhesion, 2 mL of culture medium was added to each well. The cells were further incubated for 18 h. Alamar blue assay was performed by adding Alamar blue solution to each well, incubating for 2 h, and measuring absorbance at 570 nm using an Infinite 200 Tecan automated luminometer-spectrometer. Cell counting was done by staining cells with trypan blue and using the cell counter. Statistical analysis was carried out using one-way ANOVA in IBM SPSS Statistic, with a 95% confidence interval.

## Results and Discussion

### Surface Morphology and Characteristics

**Figure 1:**
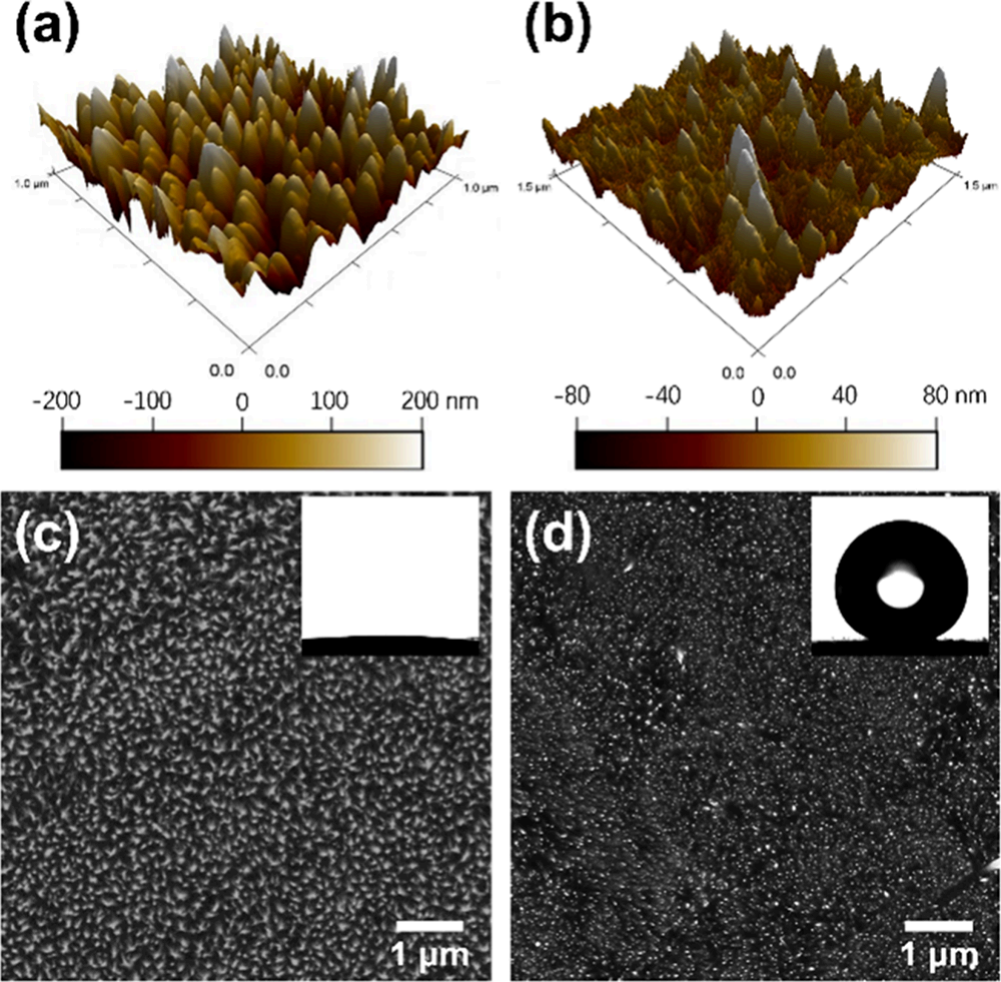
AFM images of ZnO nanopillars produced after 45 min of reaction time (a) without and (b) with PDMS coating. SEM images of the ZnO nanopillars (c) without and (d) with PDMS coating. The inset of panel (c) shows that the water contact angle is around 4.0 ± 0.5°, and the inset of panel (d) shows that the water contact angle is around 152.6 ± 2.8°.

The morphology (roughness) and interfacial energy of a surface are the key factors in controlling its wettability. Therefore, an AFM characterization of surface roughness has been carried out on ZnO nanopillar surfaces with and without PDMS coating, as shown in Figure 1a,b. The height of the nanopillars produced was mainly distributed between 350 and 400 nm with a typical diameter of 70–80 nm (Figure S2). It should also be noted that the height of the nanopillars can be controlled by adjusting the duration and the concentration of reactants during nanopillar growth. Table 1 presents a comparative analysis of surface roughness for different surface treatments; the related AFM images are shown in Figures S3 and S4. Specifically, the arithmetic average roughness (Ra) of the ZnO nanopillar surface without the PDMS coating was 58.9 ± 8.9 nm, and the root-mean-square deviation (Rq) was 73.9 ± 11.0 nm. For ZnO nanopillar surfaces coated with PDMS, their surface roughness was considerably retained, with the Ra being 39.9 ± 27.4 nm and the Rq being 49.8 ± 33.3 nm. The substantial disparity in standard deviation observed in the roughness of the PDMS-coated nanopillars can be ascribed to the thickness of the PDMS coating. Thinner PDMS layers manifest higher surface roughness, converging toward the roughness levels observed of the uncoated ZnO nanopillars. The high roughness of these nanopillar surfaces was in marked contrast to the flat zinc substrate, PDMS surface, and glass slide.

**Table 1:** Roughness of Surfaces

surface	Ra (nm)	Rq (nm)
ZnO nanopillars	58.9 ± 8.9	73.9 ± 11.0
PDMS-coated nanopillars	39.9 ± 27.4	49.8 ± 33.3
bare Zinc	9.0 ± 2.0	11.3 ± 2.6
pure PDMS	1.1 ± 0.3	1.3 ± 0.4
glass slide	3.2 ± 1.2	4.2 ± 1.8

In addition to AFM, SEM images were also captured to study the morphology of the nanopillar surfaces before and after PDMS coating. Figure 1c shows the surface of typical ZnO nanopillars prepared via a 45 min hydrothermal synthesis reaction. This provides further validation to Figure 1a that the surface was indeed composed of patterned pillar-like nanostructures. Similarly, Figure 1d reveals the pattern of the PDMS-coated nanopillar surfaces, which included a smaller quantity of pronounced nanopillars, in comparison to Figure 1c, because of the presence of the PDMS layer. Nonetheless, the outline of the nanopillars underneath is still noticeable from the SEM image, implying that the thickness of the PDMS coating was several nanometers. However, we note that the thickness of the PDMS coating could be subject to the volume and concentration of PDMS solution used in the coating process.

The crystallographic characteristics of the materials were investigated through XRD analysis, as shown in Figure S5. In the XRD spectrum of the ZnO nanopillars, a prominent diffraction peak is observed at 2θ = 34.6° (002), indicating the highly preferential growth of ZnO nanopillars perpendicular to the substrate surface. The PDMS-coated nanopillars exhibited a similar trend. Further, according to the FTIR results (Figure S6), Zn–O bond signals within the range of 500–700 cm^–1^ were identified for ZnO nanopillars fabricated by the hydrothermal synthesis reaction. On the other hand, generally, the presence of impurities corresponding to carboxylate and hydroxyl groups on the surface manifests as a series of absorption peaks spanning 1000–4000 cm^–1^ in the FTIR results. For instance, the peak at 1300–1450 cm^–1^ corresponds to O–H bending, 1400–1600 cm^–1^ corresponds to O=C–O bending, and 1700–1800 cm^–1^ corresponds to C=O stretching. In addition, the peaks at 2800–3000 and 3200–3600 cm^–1^ correspond to C–H and O–H stretching, respectively. These impurity-related peaks are notably absent in the ZnO nanopillar spectrum. The ZnO nanopillars coated with PDMS coating resultant from being exposed to 5 μL of PDMS solution during the coating process exhibit a similar trend, with slight alterations in the Zn–O bond signal, ascribed to the coating. Noteworthy is the absence of Si–CH_3_ rocking at 770–800 cm^–1^, Si–O–Si stretching at 1100–1150 cm^–1^, Si–CH_3_ bending at 1260–1380 cm^–1^, and Si–CH_3_ stretching at 2960–2975 cm^–1^ in the results of PDMS-coated ZnO nanopillars, implying a low concentration of PDMS on the ZnO pillars. To examine the effect of PDMS coating thickness on surface characteristics, FTIR tests have been conducted on the ZnO nanopillar surface with a thick PDMS coating resultant from being exposed to an excessive 25 μL, instead of 5 μL, of PDMS solution during the coating procedure. In contrast, certain signals indicative of PDMS are more readily observed on surfaces with thick coatings.

The photocatalytic activities of each surface through fluorescence intensity decay are shown in Figure S7. The bare zinc surface exhibits slow degradation of the fluorescent dye. This suggests that the exposed zinc surface, which could form an oxide layer, lacks effective photocatalytic properties against fluorescent dyes. The pure PDMS surface exhibits negligible degradation of the fluorescent dye. In contrast, ZnO nanopillars exhibit rapid photocatalytic activities, degrading the dye within 3 h. The presence of PDMS coating on the ZnO nanopillars causes the degradation of the fluorescent dye to be slower (requiring 5 h) than the uncoated ZnO nanopillars, which is still more effective than the bare zinc surface.

### Wetting Properties

Water is one of the mediums for bacteria to proliferate and spread in the initial stage of biofilm formation.^39−41^ It is thus important to understand the wetting behavior of water on the surfaces. The nanostructured surfaces have been analyzed in terms of contact angle (CA) measurements. To better demonstrate the effect of the PDMS-coated nanopillars on wettability, we measured the bare zinc, ZnO nanopillars, and PDMS-coated nanopillars. Specifically, bare zinc exhibited a static contact angle of 77.8 ± 0.6° (Figure S8), with the advancing and receding contact angles being 84.3 ± 1.7 and 46.6 ± 0.7°, respectively. For pure PDMS, the static contact angle was 106.1 ± 1.7° (Figure S9), with the advancing and receding contact angles being 110.8 ± 1.6 and 85.6 ± 0.9°, respectively. The ZnO nanopillar structure directly obtained from the hydrothermal synthesis reaction appeared superhydrophilic with a contact angle of 4.0 ± 0.5° (Figure 1c inset; Video S1) and experimentally unmeasurable contact angle hysteresis. By contrast, the ZnO nanopillar structure coated with PDMS exhibited a contact angle of 152.6 ± 2.8° (Figure 1d inset). The stability of the superhydrophobicity on the PDMS-coated ZnO nanopillars was also assessed, as shown in Figure S10. The contact angle remained essentially unchanged during a 7 day exposure. Moreover, even after an extended exposure period of 21 days, a water contact angle of around 146° was still measured. Further, the contact angle hysteresis of the PDMS-coated ZnO nanopillar surface (now denoted as superhydrophobic) was negligible, with an advancing contact angle of 154.1 ± 0.2° and a receding contact angle of 152.3 ± 0.9°, as shown in Figure S11. In addition, it is interesting to note that water droplets can roll off of the PDMS-coated nanopillar surface at a tilting angle of 1°, as shown in Figure S12 and Video S2. This remarkable superhydrophobicity with minimal tilt angle is attributed to the combination of the low-surface-energy PDMS coating and the rough ZnO nanopillars. In particular, as shown in Figure 1d, the PDMS coating was sufficiently thin to allow the surface roughness and function resulting from the ZnO nanopillars to be preserved.

**Figure 2:**
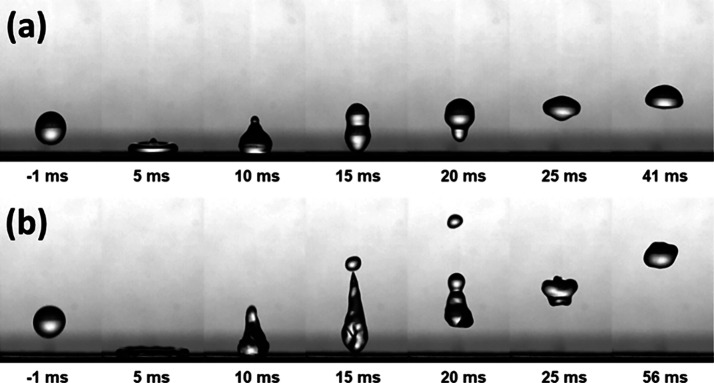
Water droplets impacting PDMS-coated nanopillar surfaces: (a) *We* = 8.62 and (b) *We* = 46.11. Droplet impact experiments were captured by a high-speed camera at a frame rate of 1000 fps.

The superhydrophobic surfaces also repelled the impact of water droplets of 2.7 mm diameter at a range of velocities. Figure 2a,b shows the morphology variations of the droplets after impacting the surfaces with different values of the Weber number (*We = D*_o_ρ*U*^2^/σ), which represents the ratio of the inertial force to the capillary force. Here, *D*_o_ is the diameter of the droplet (m), ρ is the density of the liquid (kg/m^3^), *U* is the velocity of the droplet (m/s), and σ is the interfacial tension (N/m). It is remarkable that the droplet directly rebounded from the superhydrophobic surface at a range of *We* values (*We* = 8.62 and *We* = 46.11) (Videos S3 and S4). At the beginning of the impact, the droplet recoiled to a pancake shape, which was dominated by inertial effects. This was followed by complete rebounding of the droplet from the superhydrophobic nanopillar structure. However, at a higher *We* (46.11), a satellite droplet was generated from the primary droplet. This was due to the high kinetic energy as compared to the surface tension, which caused the rim of the droplet to break up. In addition, the higher *We* also led to a higher maximum height and rebounding speed than the lower *We*. The initial rebound was followed by a series of subsequent rebounds with a gradual decrease in the impact velocity (Figure S13). By contrast, impacting droplets spread immediately against the uncoated surface of ZnO nanopillars (Figure S14a; Video S5). Similarly, the droplet did not rebound on the pure PDMS surface (Figure S14b; Video S6), although it retracted due to its hydrophobicity after spreading out upon impact. Therefore, it is deduced that the superhydrophobic nanopillar structure prevented the water droplets from spreading into the nanostructure even under substantial impalement pressure. This further implies that air pockets existed among the nanopillars coated with a thin layer of PDMS, which, as a result, repelled impacting droplets.

### Antibiofouling Properties

**Figure 3:**
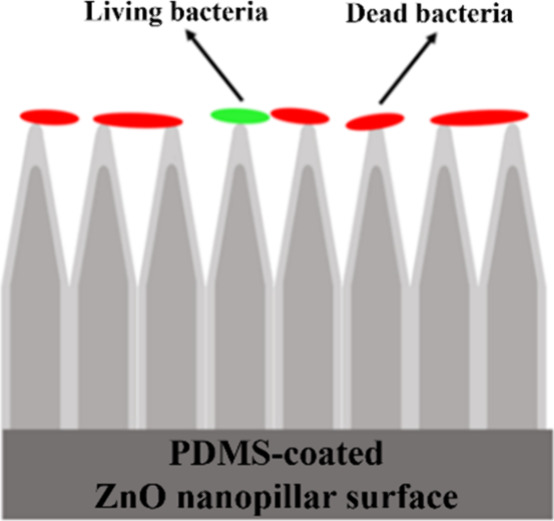
Schematic diagram of LIVE/DEAD staining experiments: dead bacteria (color of red) and living bacteria (color of green).

In order to examine the antibiofouling properties of the PDMS-coated ZnO nanopillars, bacterial viability tests were performed with dual staining kits. This allowed us to assess the viability of bacterial populations on the superhydrophobic surfaces as a function of the cell’s membrane integrity. Namely, cells that were dead or dying on the structured surfaces because of a compromised membrane stained red, whereas cells with an intact membrane appeared green, as schematically illustrated in Figure 3.

**Figure 4:**
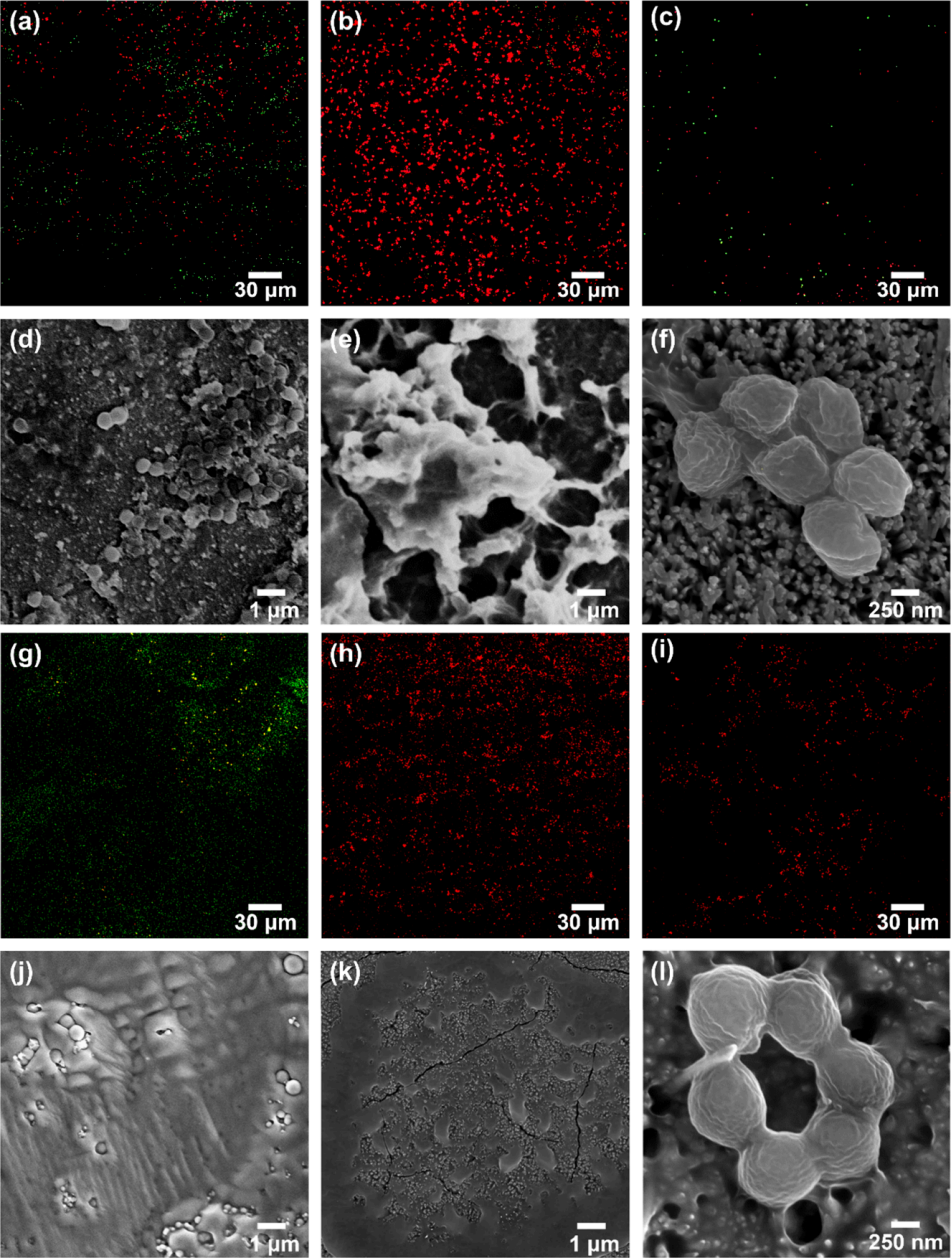
(a–c, g–i) Confocal microscopy and (d–f, j–l) SEM images of *S. aureus* after (a–f) 18 and (g–l) 72 h of incubation on different surfaces: (a, d, g, j) bare zinc, (b, e, h, k) ZnO nanopillars, and (c, f, i, l) PDMS-coated nanopillars.

The confocal microscopy images of stained *S. aureus* bacterial suspensions incubated for 18 h on the bare zinc, ZnO nanopillars, and PDMS-coated nanopillars are shown in Figure 4a–c, respectively. The confocal microscopy examination revealed that a large portion of *S. aureus* remained alive when exposed to the bare zinc surface. In contrast, the bacteria were mostly dead on the superhydrophilic ZnO nanopillar surface. However, despite the bactericidal properties, the number of dead bacteria adhering to the superhydrophilic ZnO nanopillar surface accumulated over time, as shown in Figure 4e. The antibiofouling properties of uncoated ZnO nanopillars may be compromised when bacteria cells accumulate and cover the surface, reducing the bactericidal effectiveness through direct bacteria-surface contact.^42^ In comparison, a discernible decrease in the bacterial presence was observed on the PDMS-coated nanopillar surface, although a coexistence of live and dead bacteria persisted. Nonetheless, the coverage of bacteria was less on the superhydrophobic ZnO nanopillars, likely due to the low adhesion, than on the bare zinc and untreated ZnO nanopillars. SEM images of *S. aureus* on the bare zinc, ZnO nanopillars, and PDMS-coated nanopillars are shown in Figure 4d–f. It is evident that *S. aureus* initiated the formation of biofilm on the bare zinc surface following an 18 h incubation. Bacterial remnants that were deformed/dehydrated were observed on the ZnO nanopillars. This is consistent with the confocal microscopy analysis that showed the nonviability of the bacterial population of *S. aureus* on the uncoated ZnO nanopillars. By contrast, there was an apparent absence of large bacterial populations of *S. aureus* on the superhydrophobic ZnO nanopillars. From the SEM study, there were sparsely distributed bacteria of *S. aureus* on the PDMS-coated ZnO nanopillars, similar to the confocal microscopy result. A closer observation (Figure 4f) indicates that some of the bacteria appeared to be structurally deformed after being exposed to the PDMS-coated ZnO nanopillars.

Tests were also conducted with an incubation time of 72 h to examine whether the antibiofouling effect could be sustained on the surfaces for longer durations. The confocal microscopy and SEM images reveal that on the bare zinc surface, *S. aureus* cells were still able to survive and form biofilms (Figure 4g,j). In contrast, the ZnO nanopillars continued to exhibit similar trends to the 18 h incubation, with an extensive coverage of dead bacteria, as shown in Figure 4h,k. On the superhydrophobic ZnO nanopillars coated with PDMS, the longer incubation of 72 h allowed the accumulation of *S. aureus* to moderately increase, as shown in Figure 4i,l, in comparison with the 18 h incubation. Nevertheless, the coverage of bacteria after 72 h of incubation on the superhydrophobic PDMS-coated ZnO surface was still less than on the uncoated ZnO surface. Also, the bacteria that were residual on the superhydrophobic surface appeared to be largely dead (Figure 4i). This is attributed to the extended exposure to the photocatalytic activities of ZnO underneath the surface that caused the bacteria to be structurally deformed (Figure 4l).

**Figure 5:**
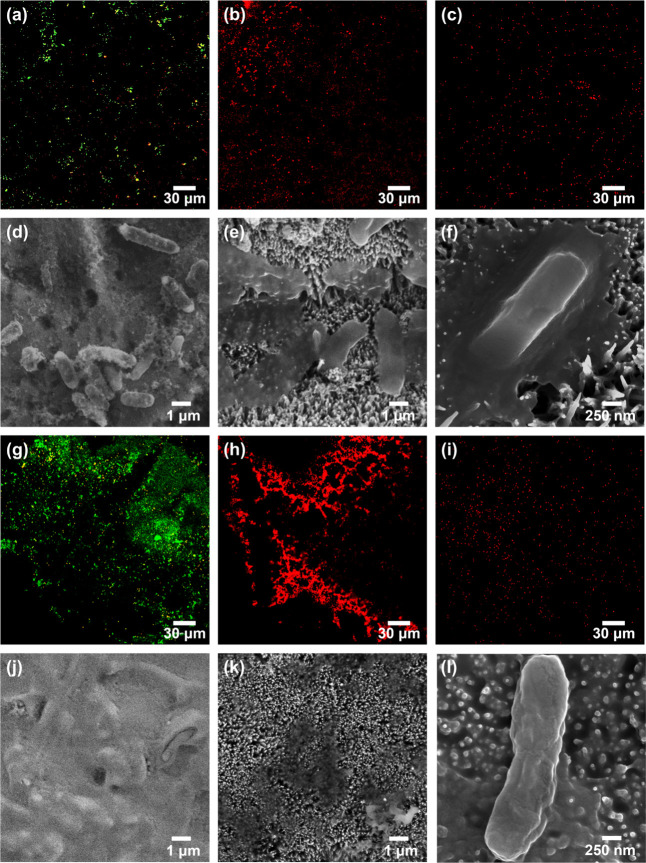
(a–c, g–i) Confocal microscopy and (d–f, j–l) SEM images of *E. coli* after (a–f) 18 and (g–l) 72 h of incubation on different surfaces: (a, d, g, j) bare zinc, (b, e, h, k) ZnO nanopillars, and (c, f, i, l) PDMS-coated nanopillars.

Furthermore, the confocal microscopy images (Figure 5a–c) show stained *E. coli* bacterial suspensions incubated for 18 h on different surfaces: the bare zinc, ZnO nanopillars, and PDMS-coated nanopillars. Similar to the findings with *S. aureus*, most bacteria of *E. coli* were green stained on the bare zinc surface, whereas nearly all bacterial populations appeared red stained on the ZnO nanopillars. However, on PDMS-coated nanopillars, the stained *E. coli* emitted red signals indicative of dead cells. This is attributed to the disparities in the bacterial morphology and inner structure between *S. aureus* and *E. coli*. Generally, *S. aureus* is usually spherically shaped, with a diameter of approximately 0.5–1.0 μm and a thicker peptidoglycan layer than *E. coli*. In contrast, *E. coli* is typically rod-shaped, with a width of 0.5 μm and a length of 1.5 μm.^43−45^ The differences in structural morphology between the bacteria may have had an effect on their viability on nanopillar-based structures. The shape of the *E. coli* bacteria could have had greater surface contact with the nanopillar structures than that of *S. aureus*, even in the presence of the PDMS layer. This would impose a stressful stimulus on bacterial cell walls, causing irreparable cell wall rupture and bacterial death.^46^ Indeed, SEM images (Figure 5d–f) further illustrate that bacteria of *E. coli* with disfigured appearances were observed on both the uncoated ZnO nanopillars (Figure 5e) and the PDMS-coated ZnO nanopillars (Figure 5f). This was more notable on the uncoated ZnO nanopillar structure, which could cause the bacteria to be punctured as adhesive forces pulled them toward the surface due to its superhydrophilicity.^24^ Nevertheless, the PDMS-coated ZnO nanopillars, because of the low interfacial adhesion associated with the superhydrophobicity, reduced the attachment of *E. coli* on the surface.

The 72 h incubation test was also performed for *E. coli*, with confocal microscopy and SEM images collected to analyze the behavior of *E. coli* on different surfaces. Bacterial cells on bare zinc still exhibited green signals and displayed a tendency to form large-area biofilms (Figure 5g). SEM observations confirmed the presence of some large-area biofilms on the surface, as shown in Figure 5j. In contrast, the trend observed after the 72 h incubation of the uncoated ZnO nanopillars and the superhydrophobic ZnO nanopillars remained essentially consistent with that observed after the 18 h incubation. It is worth noting that in addition to the results of the above three kinds of surfaces, confocal microscopy and SEM images of the control group, glass, and pure PDMS were also collected at different incubation times (18 and 72 h), as shown in Figures S15–S17. We note that the term “control” in this work refers to tests wherein the bacteria are exposed to conditions without any specific surface material. From the results, it can be observed that bacteria could proliferate in the control group and remained active. Similarly, as neither glass nor PDMS possessed inherent antibiofouling properties, they also allowed substantial proliferation and biofilm formation to take place. Therefore, bacteria on the surfaces of ZnO nanopillars with excessive PDMS coatings did not exhibit any noticeable deformation (Figures S16 and S17).

**Figure 6:**
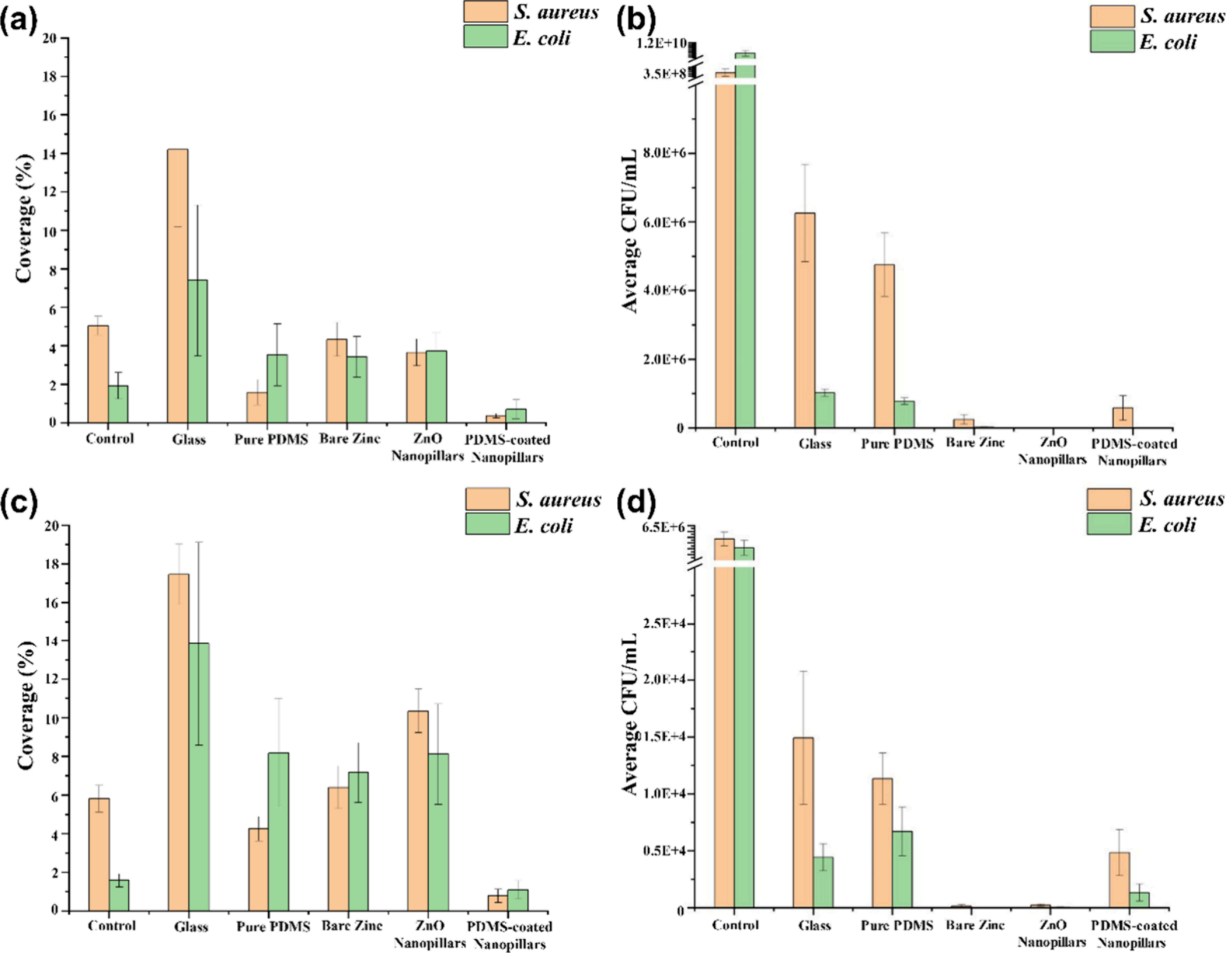
Coverage percentage of bacteria population after (a) 18 and (c) 72 h incubation. The number of colony forming units (CFU)/mL on each surface sample after (b) 18 and (d) 72 h incubation.

The coverage percentage of bacteria populations on various surfaces was calculated based on the confocal microscopy images, as shown in Figure 6a,c corresponding to the 18 h and 72 h of incubation, respectively.^47^ A consistent trend is observed, where the bacterial coverage on superhydrophobic PDMS-coated nanopillars is significantly reduced. This is attributed to the low liquid–solid adhesion associated with the superhydrophobicity of the surfaces that were characterized by the small contact angle hysteresis and tilting angle. Additionally, the antimicrobial properties of the surfaces are quantitatively illustrated in Figure 6b,d, using colony-forming units (CFU). Notably, there was very few CFU that could be detected from the samples, which had been exposed to the superhydrophilic ZnO nanopillars in both the cases of 18 h and 72 h incubations. This is consistent with recent studies that nanostructures of ZnO can exhibit antimicrobial activities through bacterial cell wall damage.^48,49^ In particular, a recent study used a similar approach to the development of ZnO nanopillars coated with PDMS for antibacterial applications.^50^ It is noteworthy that in our case, the ZnO nanopillars exhibited more uniform upright morphologies, which had been preserved even in the presence of PDMS coatings (Figure 1d). This makes it possible to enhance the antimicrobial properties of the surfaces through a combination of the two following complementary mechanisms: (a) direct bacterial membrane puncture and (b) photocatalytic activities of ZnO. However, there is a possibility that PDMS layers exceeding a certain thickness, typically several nanometres, could impede or slow down the photocatalytic activity.^34,51^ Thicker PDMS layers also obscure the nanopillar structure, hindering the mechanical stress of the pillars against the bacteria. This will affect the surface’s antibiofouling behaviors, as shown in Figures S16, S17, and S19.

On the other hand, the number of CFU on ZnO nanopillars is significantly lower than those on glass and pure PDMS. Further, we note that the low intensity of the CFU of the bare zinc surface is ascribed to the arbitrary presence of an oxide layer on the zinc surface, which could lead to antibiofouling behaviors. However, given that live bacteria signals can be widely found on the zinc surfaces under confocal microscopy, they are not considered to be resistant to biofouling. Furthermore, to evaluate the efficacy of superinfection prevention, multicycle tests were implemented (Figures S20 and S21). The antibacterial response and behavior of each sample type over the multiple cycles of testing aligned consistently with the trends observed in the initial antibiofouling efficacy testing. In addition, the relevant surfaces showed no signs of cytotoxicity against mammalian cells (Figure S22), indicating good biocompatibility.

## Conclusions

In conclusion, ZnO nanopillars were coated with PDMS to form superhydrophobic surfaces with antibiofouling properties. Droplets of water on these superhydrophobic surfaces exhibited large apparent contact angles and low contact angle hysteresis of within 3°. In addition, such surfaces showed low droplet adhesion, characterized by a low tilting angle of 1°. The liquid repellence of the surfaces was further demonstrated by droplet impingement tests, where impinging droplets bounced off over a range of Weber numbers (8–46). Notably, the PDMS-coated ZnO nanopillars exhibited effective antibiofouling behaviors under ambient illumination due to the intrinsic photocatalytic activities of ZnO nanomaterials. Therefore, the superhydrophobic PDMS-coated ZnO surfaces integrated a low interfacial adhesion and bactericidal effect. This synergy, without requiring additional UV illumination or antimicrobial agents, makes the PDMS-coated ZnO nanopillar surfaces a promising material for antibiofouling applications.

## Supporting Information Available

Formation mechanism of ZnO nanopillar, agar plate preparation and antibiofouling efficacy testing, surface morphology, X-ray diffraction (XRD) analysis, Fourier transform infrared (FTIR) spectroscopy, photocatalysis, wetting properties, Weber number calculation, droplet impact, antibiofouling properties, multicycle test, cytotoxicity tests, and bacterial coverage (PDF)
Droplet contact angle on uncoated ZnO nanopillars (MP4)
Droplet roll-off on PDMS-coated ZnO nanopillars (MP4)
Droplet impact on PDMS-coated ZnO nanopillars with Weber number = 8.62 (MP4)
Droplet impact on PDMS-coated ZnO nanopillars with Weber number = 46.11 (MP4)
Droplet impact on uncoated ZnO nanopillars (MP4)
Droplet impact on pure PDMS (MP4)
