# Supplementary material for: Wettability and Bactericidal Properties of Bioinspired ZnO Nanopillar Surfaces
Source: Langmuir. 2024 Mar 27;40(14):7353–63. doi: 10.1021/acs.langmuir.3c03537 (PMC11008234; doi:10.1021/acs.langmuir.3c03537)
Supplement: Supplementary file 1 — la3c03537_si_001.pdf [file la3c03537_si_001.pdf]

Wettability and Bactericidal Properties of Bioinspired ZnO Nanopillar Surfaces

Jitao Zhang[a], *Georgia Williams*[b], *Thanaphun Jitniyom*[a], *Navdeep Sangeet Singh*[a], *Alexander Saal*[a], *Lily Riordan*[c], *Madeline Berrow*[c], *James Churm*[a], *Manuel Banzhaf*[b#*], *Felicity de Cogan*[c*], *Nan Gao*[a*]

[a] School of Engineering, University of Birmingham, Edgbaston, Birmingham, B15 2TT, United Kingdom

[b] School of Biosciences, University of Birmingham, Edgbaston, Birmingham, B15 2TT, United Kingdom

[c] School of Pharmacy, University of Nottingham, University Park, Nottingham, NG7 2RD, United Kingdom

[*] Email: n.gao@bham.ac.uk

[*] Email: Felicity.DeCogan@nottingham.ac.uk

[*] Email: m.banzhaf@bham.ac.uk

SUPPLEMENTARY RESULTS

S1 Formation mechanism of ZnO nanopillars

The formation of ZnO pillars on bare zinc substrate in an alkaline zincate solution (NaOH + Zn(NO3)2·6H2O) can be described by the following reactions:

$$\mathrm{Zn} + 2\mathrm{H_2O} + 2\mathrm{OH^-} \rightarrow \mathrm{Zn(OH)_4^{2-}} + \mathrm{H_2} \uparrow$$

$$\mathrm{Zn(OH)_4^{2-}} = \mathrm{ZnO} \downarrow + \mathrm{H_2O} + 2\mathrm{OH^-}$$

Throughout the process of ZnO nanopillar growth, zinc atoms dissolve into the solution, leading to an increased concentration gradient of zincate ions near the bare zinc substrate. Consequently, the growth rate along the z-axis (i.e., the (001) direction according to the XRD results shown in Section S4) gradually decreases from the root to the top of the nanostructure, ultimately resulting in the formation of nanopillars.

S2 Agar plates preparation and anti-biofouling efficacy testing

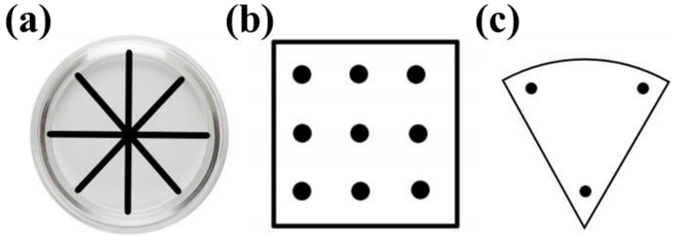

Figure S1. Schematic diagram of agar plates preparation and anti-biofouling efficacy testing: (a) Agar plate with divided areas. (b) Bacterial culture spots on surface. (c) Bacterial drops on an individual section of the agar plate.

S3 Surface morphology

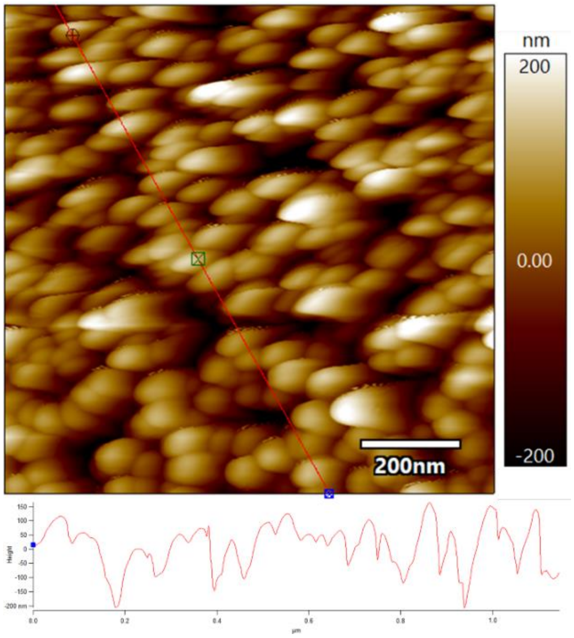

Figure S2. AFM image of ZnO nanopillars.

Table S1. Surface roughness of plain surfaces

**Ra (nm)**	Bare zinc	Glass	Pure PDMS
1st	7.19	2.08	0.97
2nd	8.69	3.01	0.90
3rd	11.12	4.54	1.43
Average	9.00 ± 1.98	3.21 ± 1.24	1.10 ± 0.29
**Rq (nm)**	Bare zinc	Glass	Pure PDMS
1st	9.07	2.72	1.13
2nd	10.72	3.74	1.07
3rd	14.14	6.21	1.70
Average	11.31 ± 2.59	4.22 ± 1.79	1.30 ± 0.35

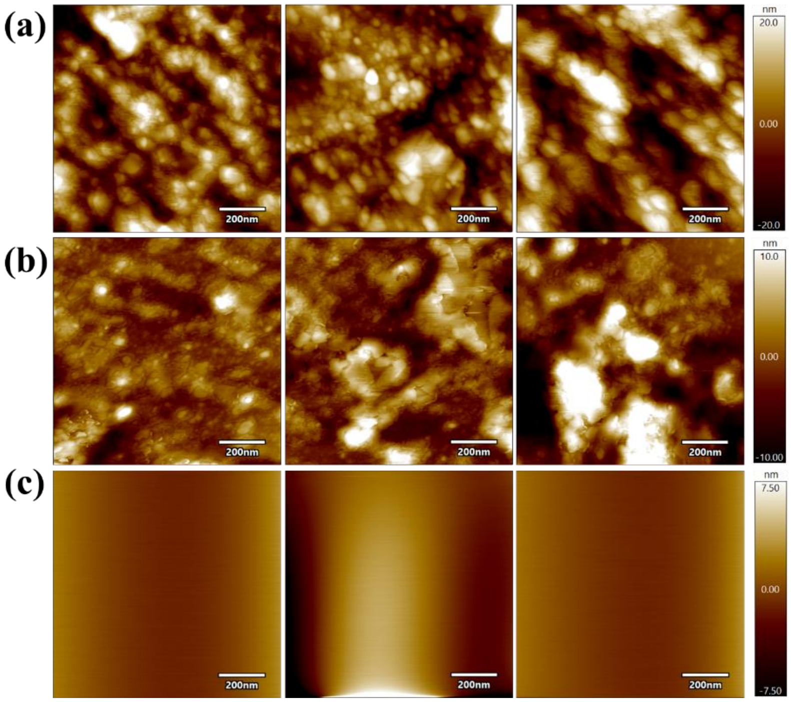

Figure S3. AFM images of the plain surfaces used in roughness calculation: (a) Bare zinc, (b) Glass and (c) Pure PDMS

Table S2. Surface roughness of nanopillar-based surfaces

**Ra (nm)**	ZnO nanopillars	PDMS-coated nanopillars
1st	64.47	17.08
2nd	65.05	15.95
3rd	56.30	12.89
4th	67.61	72.68
5th	56.16	60.95
6th	43.65	60.04
Average	58.87 ± 8.85	39.93 ± 27.38
**Rq (nm)**	ZnO nanopillars	PDMS-coated nanopillars
1st	80.67	21.33
2nd	82.42	21.45
3rd	70.80	16.64
4th	84.06	88.82
5th	70.26	76.08
6th	54.95	74.73
Average	73.86 ± 10.99	49.84 ± 33.31

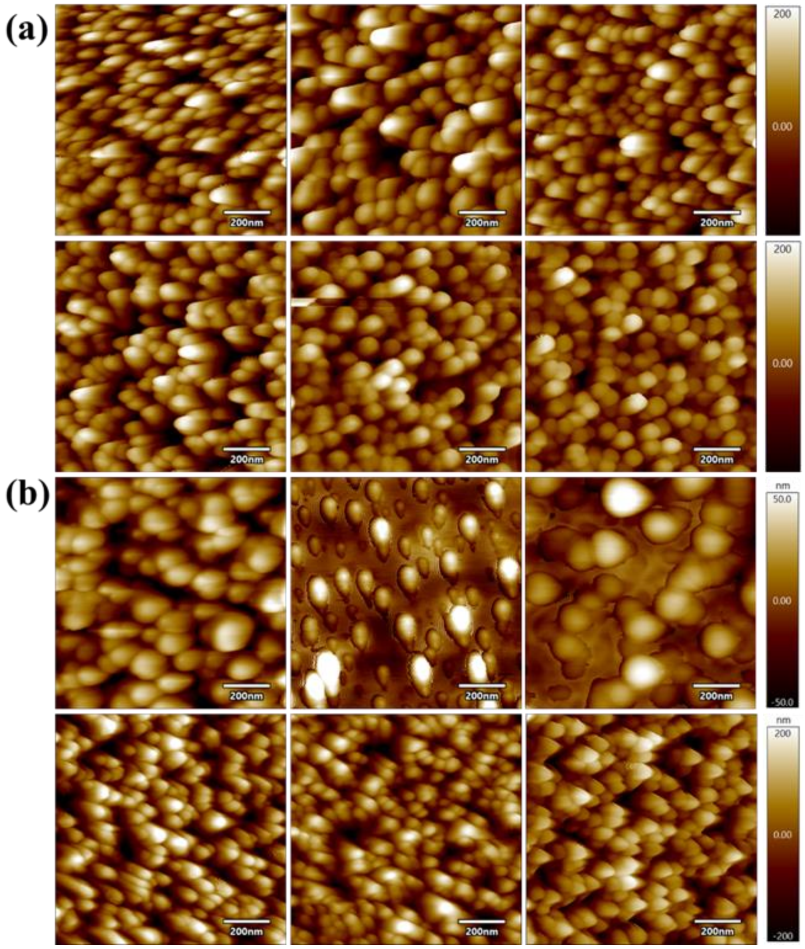

Figure S4. AFM images of nanopillar-based surfaces used in roughness calculation: (a) ZnO nanopillars and (b) PDMS-coated ZnO nanopillars.

S4 X-ray diffraction (XRD) analysis

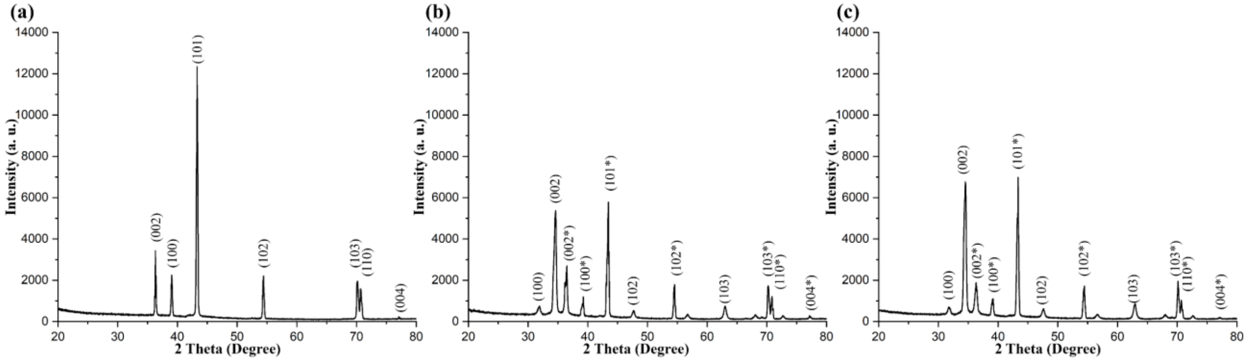

Figure S5. (a) Bare zinc, (b) ZnO nanopillars and (c) PDMS-coated nanopillars. Peaks marked with asterisks (*) are attributed to the zinc substrate.

S5 Fourier-transform infrared (FTIR) spectroscopy

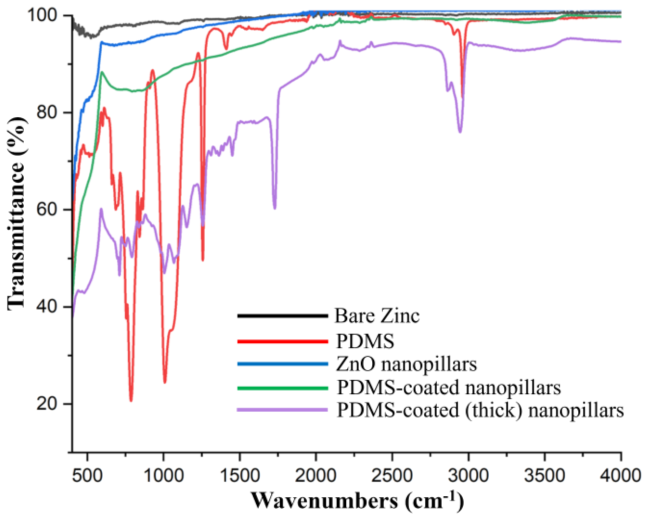

Figure S6. The FTIR spectra of samples.

S6 Photocatalysis

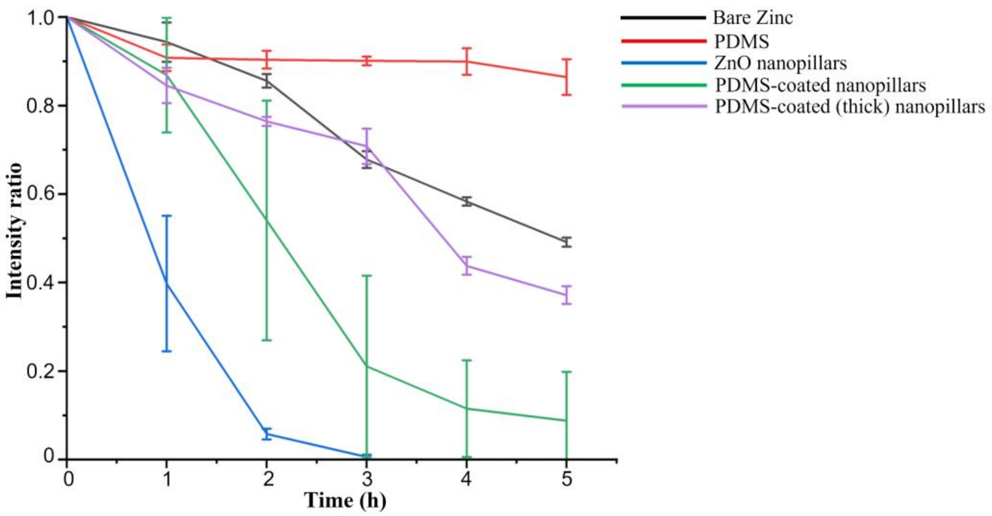

Figure S7. Fluorescence intensity changes of CF633 measured by UV-visible spectroscopy.

S7 Wetting properties

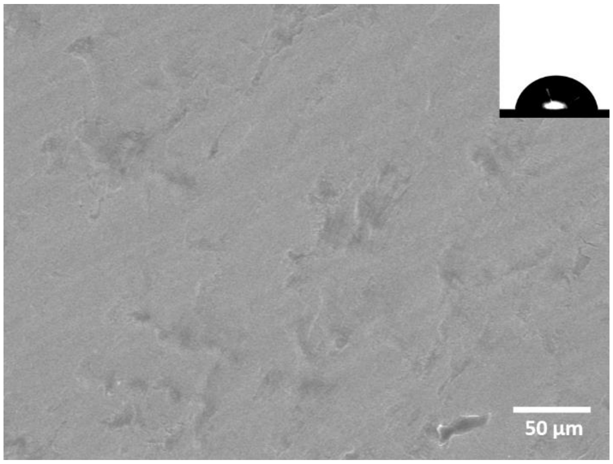

Figure S8. SEM image of bare zinc. The inset shows a water droplet on the bare zinc, with a static contact angle of around 77.8 ± 0.6°.

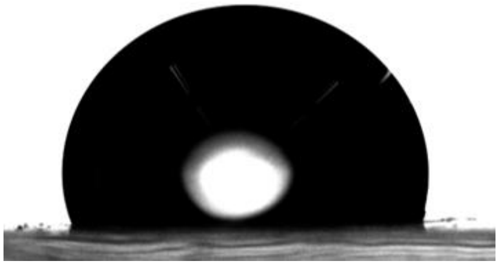

Figure S9. Water droplet on pure PDMS, with a static contact angle of around 106.1 ± 1.7°.

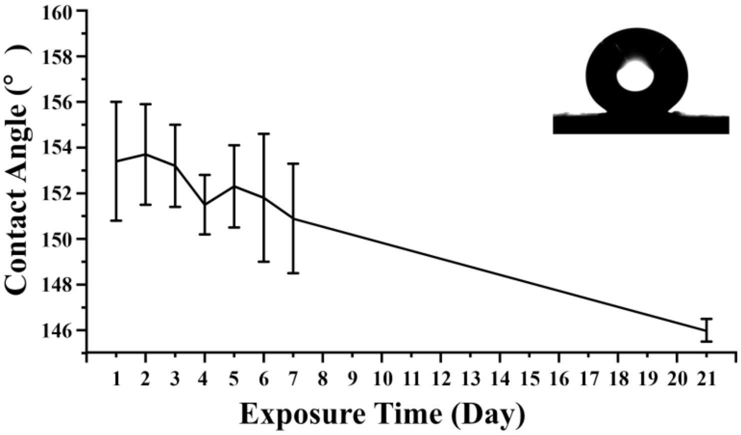

Figure S10. Droplet contact angle as a function of time. The inset shows that the water contact angle was around 146 ± 0.5° even after 21 days.

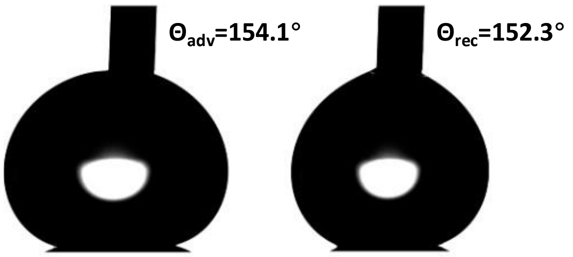

Figure S11. Measurement of dynamic contact angles of water on PDMS-coated ZnO nanopillars.

Table S3. Dynamic contact angles of PDMS-coated nanopillars

Frame No.	Advancing contact angle (°)	Receding contact angle(°)
1	153.9	153.3
2	153.8	153.3
3	154.3	153.3
4	154.3	153.1
5	154.3	152.7
6	154.3	152.2
7	154.1	151.7
8	154.0	151.4
9	153.9	151.5
10	153.8	151.2
Average	154.1 ± 0.2	152.3 ± 0.9

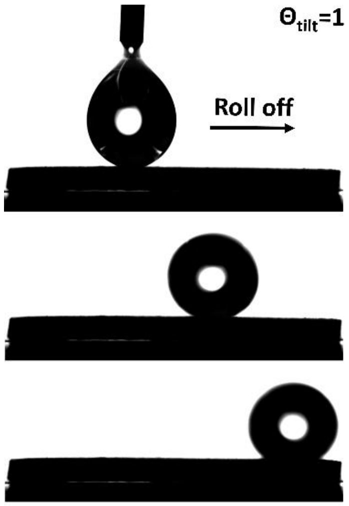

Figure S12. Water droplet rolling off.

S8 Weber number calculation

Weber number is calculated using the below formula:

$$We = \frac{D_o \rho U^2}{\sigma}$$

D_o is the diameter of the droplet, ρ is the density of the liquid (ρ = 998 kg/m^3 for water), U is the velocity of the droplet and σ is the interfacial tension (σ = 0.072 N/m for water).

The dimension calibration, as shown in Figure S13(a) and Table S4, has been used to calculate the diameter of the droplet and the velocity. The size and pixel numbers are compared in Table S4, using the black screw in the image left as a reference system. Calculation details for Weber numbers are listed in Table S5. We perform calculations using the data from a single frame captured just before the droplet hits the surface (-1 ms). Figure S13(b) and (c) also show the full bouncing series and velocity change of droplet impact on PDMS-coated superhydrophobic surfaces.

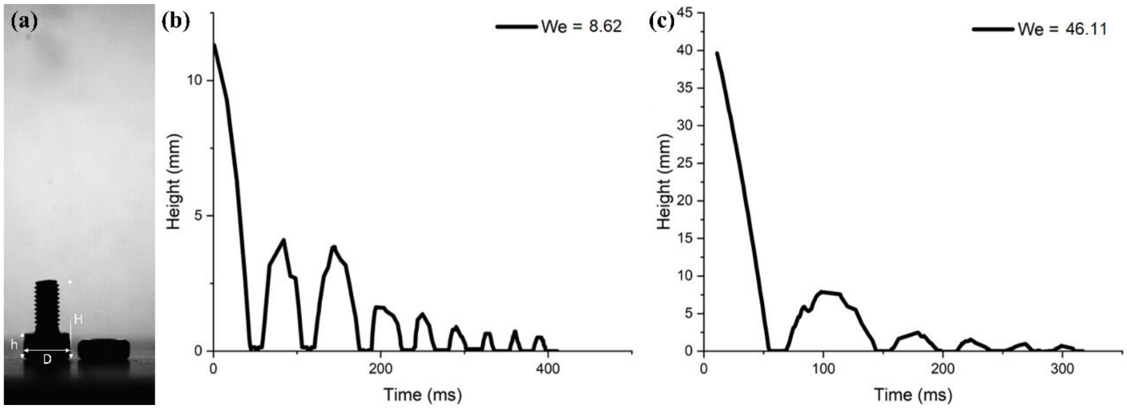

Figure S13. (a) Dimension calibration: 1 pixel in the image should be equal to 0.037 mm. Full bouncing series and velocity change of a water droplet (D_o = 2.7 mm) on superhydrophobic PDMS-coated nanopillars with (b) *We* = 8.62 and (c) *We* = 46.11.

Table S4. Dimension calibration.

	Screw total height (H)	Screw head diameter (D)	Screw head thickness (h)
Real size (mm)	9	5.45	3
Pixels	250	148	79
Converting ratio	0.036 mm/pixel	0.037 mm/pixel	0.038 mm/pixel
The converting ratio used in the calculation = 0.037 ± 0.001 mm/pixel (average)			

Table S5. Weber number calculation.

	Impact time (Frame)	Impact distance (Pixel)	Converted impact time (ms)	Converted impact distance (mm)	Impact velocity calculation (m/s)	Weber number
Impact 1	1	13	1	0.48	0.48	8.62
Impact 2	1	30	1	1.11	1.11	46.11

S9 Droplet impact

The droplet impact images of the superhydrophilic ZnO nanopillars and pure PDMS surfaces are shown in Figure S14.

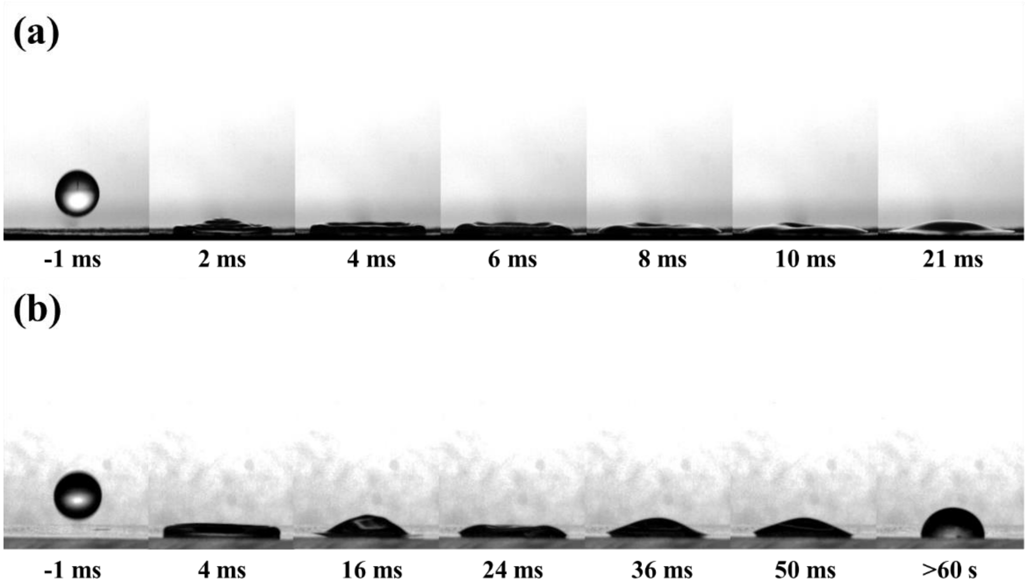

Figure S14. Water droplet impacting on (a) uncoated ZnO nanopillar surfaces and (b) pure PDMS surface with *We* = 46.11.

S10 Anti-biofouling properties

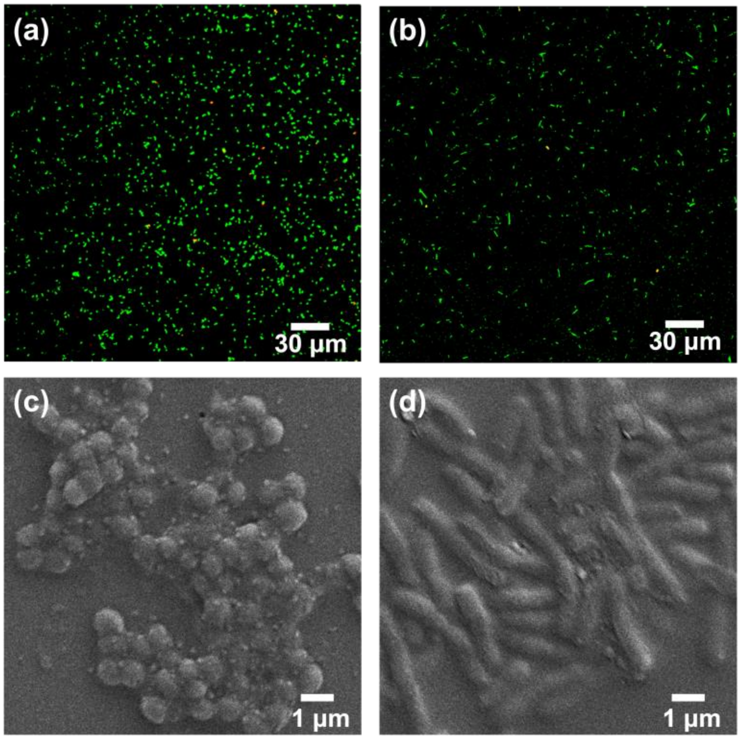

Figure S15. Confocal microscopy (a, b) and SEM (c, d) images of bacteria from the "no surface" control group: *S. aureus* (a, c) and *E. coli* (b, d).

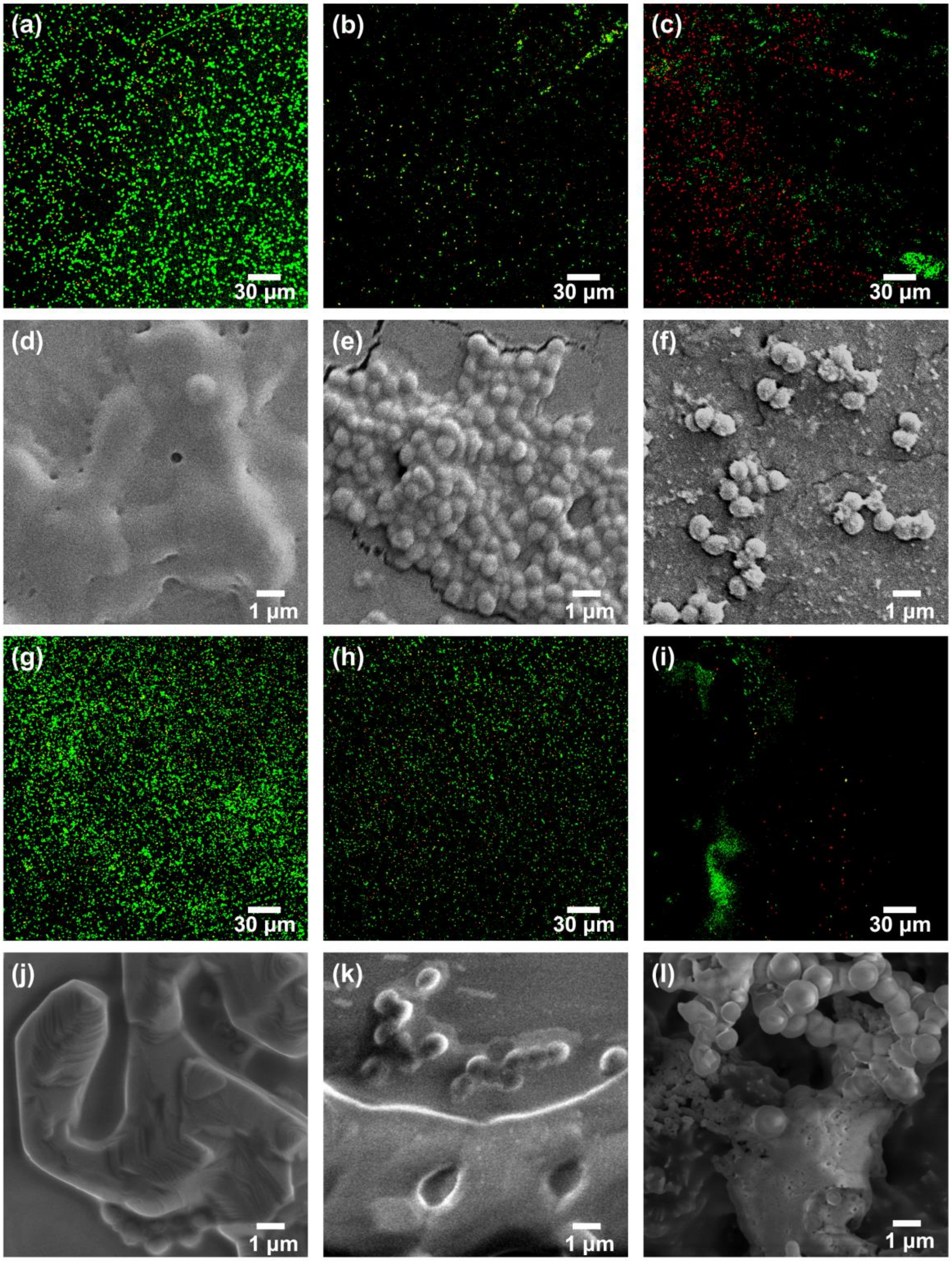

Figure S16. Confocal microscopy (a-c and g-i) and SEM (d-f and j-l) images of *S. aureus* after 18 (a-f) and 72 (g-l) hours of incubation, respectively, on different surfaces: glass (a, d, g, j), pure PDMS (b, e, h, k) and thick PDMS-coated nanopillars (c, f, i, l).

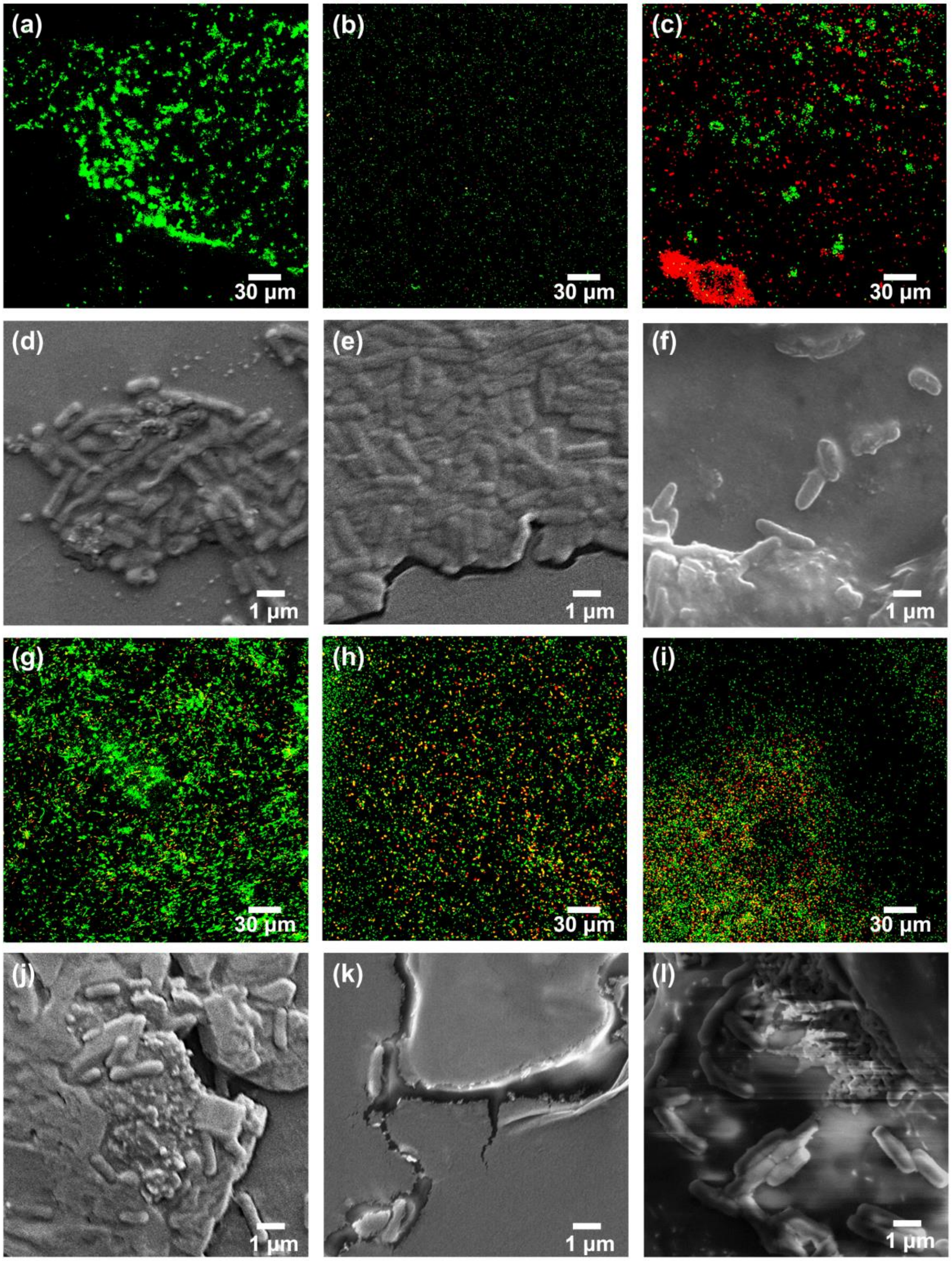

Figure S17. Confocal microscopy (a-c and g-i) and SEM (d-f and j-l) images of *E. coli* after 18 (a-f) and 72 (g-l) hours of incubation, respectively, on different surfaces: glass (a, d, g, j), pure PDMS (b, e, h, k) and thick PDMS-coated nanopillars (c, f, i, l).

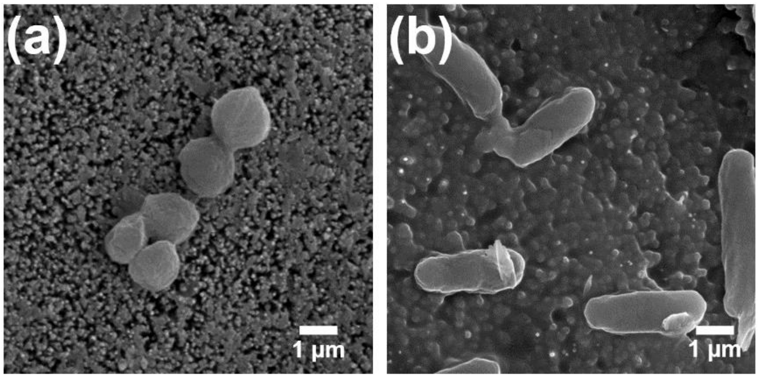

Figure S18. SEM image of (a) *S. aureus* and (b) *E.coli* on PDMS-coated nanopillars.

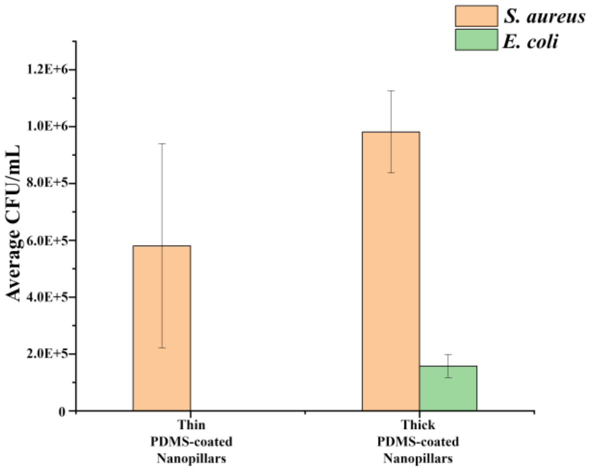

Figure S19. The number of colony forming units (CFU)/mL on different PDMS-coated nanopillar surfaces after 18-hour incubation.

S11 Multi-cycle tests

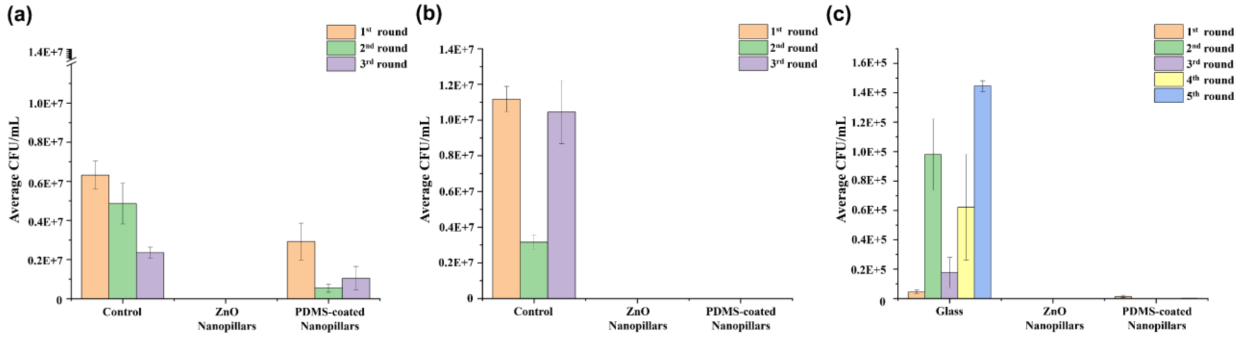

Figure S20. The number of colony forming units (CFU)/mL on the ZnO nanopillar-based surfaces of (a) *S. aureus* after three rounds of 18-hour incubation and *E. coli* after (b) three rounds and (c) five rounds of 18-hour incubation.

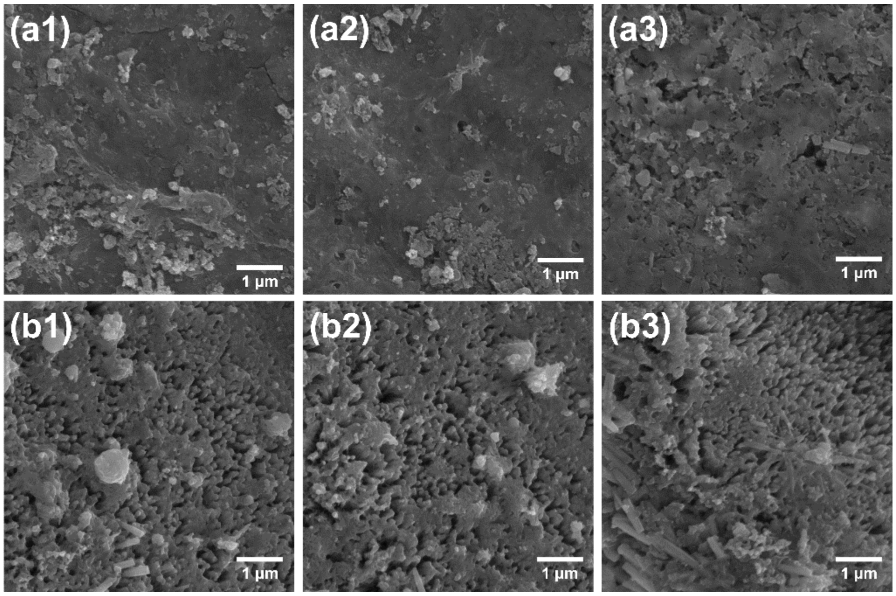

Figure S21. SEM images of surface morphology after multi-cycle tests: (a) ZnO nanopillars and (b) PDMS-coated ZnO nanopillars.

S12 Cytotoxicity tests

After the anti-biofouling efficacy tests, a cytotoxicity test was conducted to probe the safety and biocompatibility of the relevant surfaces. The results indicated no statistical significance (p=0.247) in the absorbance at 570 nm between the cell culture media and the relevant surfaces, as shown in Figure S22. This implies that the metabolism of resazurin in the Alamar blue solution was not significantly different, and consequently, the viability of the 3T3 fibroblast cells remained unaffected. The treated surfaces exhibited no indication of cytotoxicity against our mammalian cells.

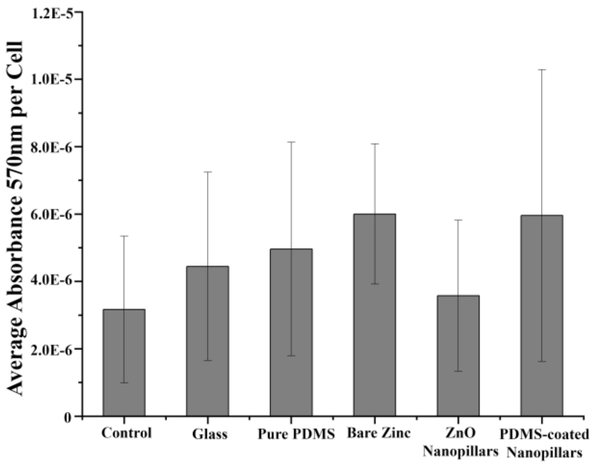

Figure S22. Cytotoxicity results from different surfaces.

S13 Bacterial coverage

The bacterial coverage on surfaces was characterised utilizing FIJI (ImageJ). In this process, confocal microscopy images were imported into FIJI and subsequently converted to 8-bit images. The pixel intensity distribution in this process was visualized, enabling the identification of selected pixels or regions of interest, which were highlighted in white/grey. Subsequent image analysis included the determination of bacteria count, total area, average size, and coverage area. Figure S23 provides an illustrative example of FIJI's capability in detecting bacteria attached to sample surfaces from confocal microscopy images. Tables S6 and S7 present coverage data derived from the confocal microscopy images. The confocal microscopy images utilized for this analytical process are displayed in Figures S24-27.

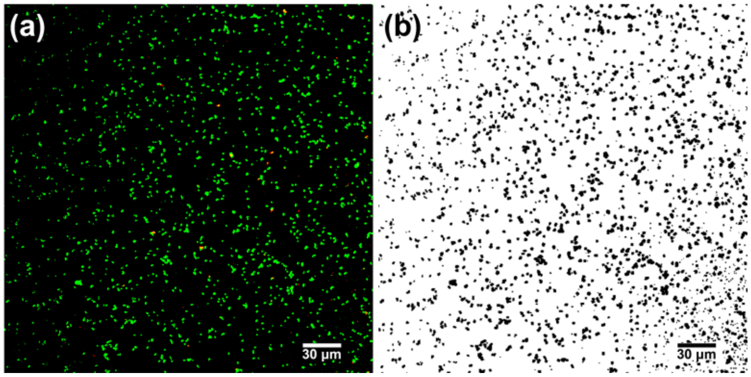

Figure S23. Illustrative example of bacteria characterisation based on confocal microscopy images: (a) Original confocal microscopy image and (b) FIJI converted image.

Table S6. Bacterial coverage (18-hour incubation).

***S. aureus* (%)**	Image 1	Image 2	Image 3	Image 4	Image 5	Average
Control	4.23	5.16	5.42	5.40	5.02	5.05±0.49
Glass	15.75	10.75	20.56	11.83	12.08	14.19±4.03
Pure PDMS	2.08	0.91	2.03	0.81	2.06	1.58±0.66
Bare zinc	3.72	3.73	4.68	5.75	3.83	4.34±0.88
ZnO nanopillars	4.87	3.50	3.44	3.36	3.11	3.66±0.69
PDMS-coated nanopillars	0.36	0.18	0.42	0.37	0.50	0.37±0.12
***E. coli* (%)**	Image 1	Image 2	Image 3	Image 4	Image 5	Average
Control	1.69	1.72	1.26	3.08	1.87	1.92±0.68
Glass	9.06	10.53	11.03	3.08	3.27	7.39±3.92
Pure PDMS	2.75	3.98	2.68	6.14	2.11	3.53±1.61
Bare zinc	1.97	2.79	4.35	3.51	4.49	3.42±1.06
ZnO nanopillars	2.49	4.29	5.05	3.40	3.37	3.72±0.98
PDMS-coated nanopillars	1.09	0.24	1.30	0.21	0.68	0.70±0.49

Table S7. Bacterial coverage (72-hour incubation).

***S. aureus* (%)**	Image 1	Image 2	Image 3	Image 4	Image 5	Average
Control	3.97	5.18	4.08	4.49	6.33	5.81±0.69
Glass	16.26	16.06	18.69	19.53	16.73	17.45±1.56
Pure PDMS	5.30	4.11	3.74	3.79	4.33	4.25±0.63
Bare zinc	5.38	5.97	5.84	8.21	6.57	6.39±1.10
ZnO nanopillars	11.86	11.91	11.12	11.85	11.02	10.35±1.13
PDMS-coated nanopillars	1.19	0.40	1.04	0.49	0.80	0.78±0.34
***E. coli* (%)**	Image 1	Image 2	Image 3	Image 4	Image 5	Average
Control	1.71	1.51	1.61	1.08	2.01	1.58±0.34
Glass	18.18	11.91	9.98	20.59	8.55	13.84±5.27
Pure PDMS	12.25	9.86	5.80	7.24	5.69	8.17±2.83
Bare zinc	6.57	5.66	7.60	9.62	6.35	7.16±1.54
ZnO nanopillars	9.19	9.78	5.48	5.23	10.92	8.12±2.60
PDMS-coated nanopillars	1.22	0.80	1.44	0.42	1.59	1.09±0.48

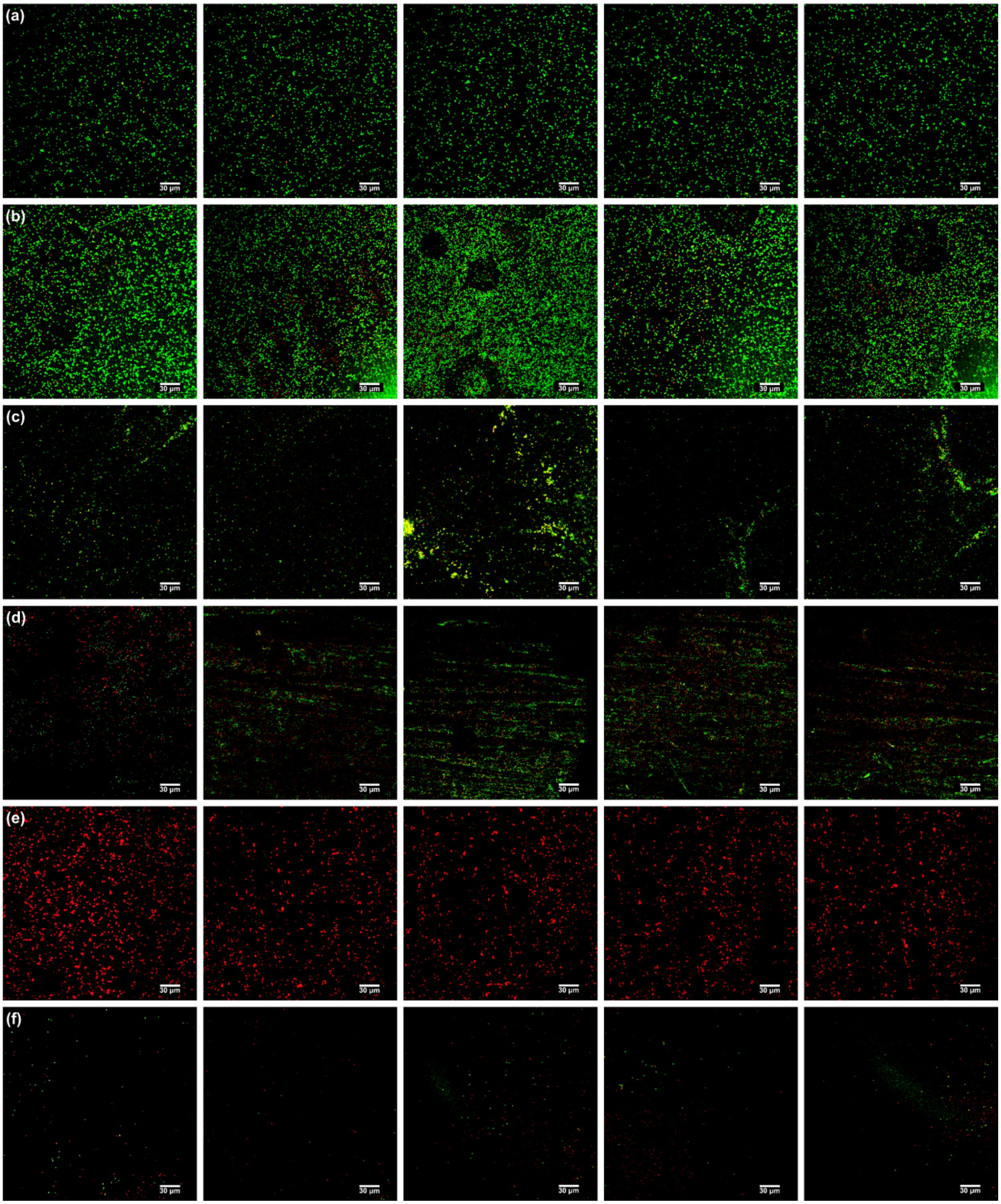

Figure S24. Confocal microscopy images of *S. aureus* after 18 hours of incubation on (a) control, (b) glass, (c) pure PDMS, (d) bare zinc, (e) ZnO nanopillars, (f) PDMS-coated nanopillars.

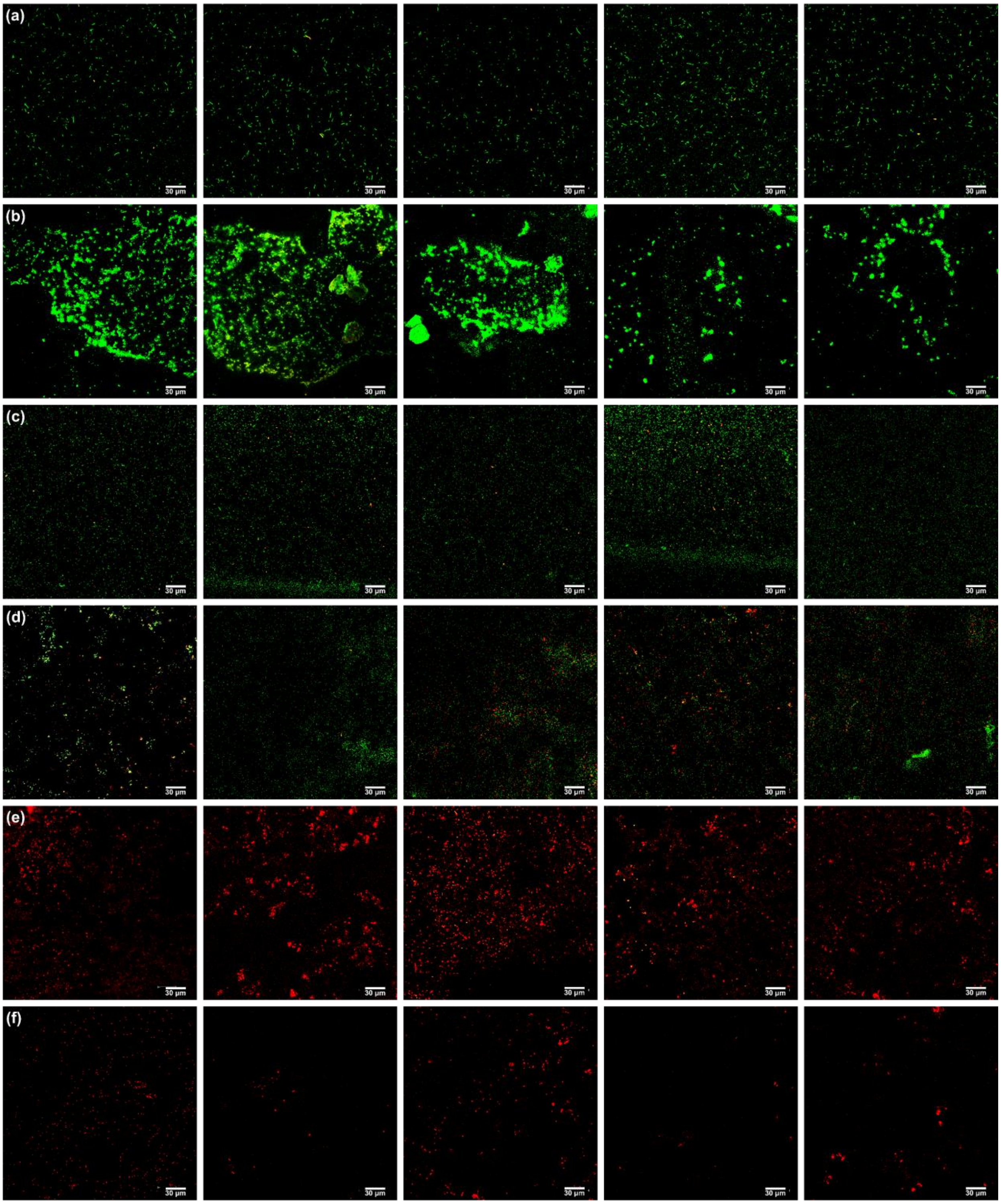

Figure S25. Confocal microscopy images of *E. coli* after 18 hours of incubation on (a) control, (b) glass, (c) pure PDMS, (d) bare zinc, (e) ZnO nanopillars, (f) PDMS-coated nanopillars.

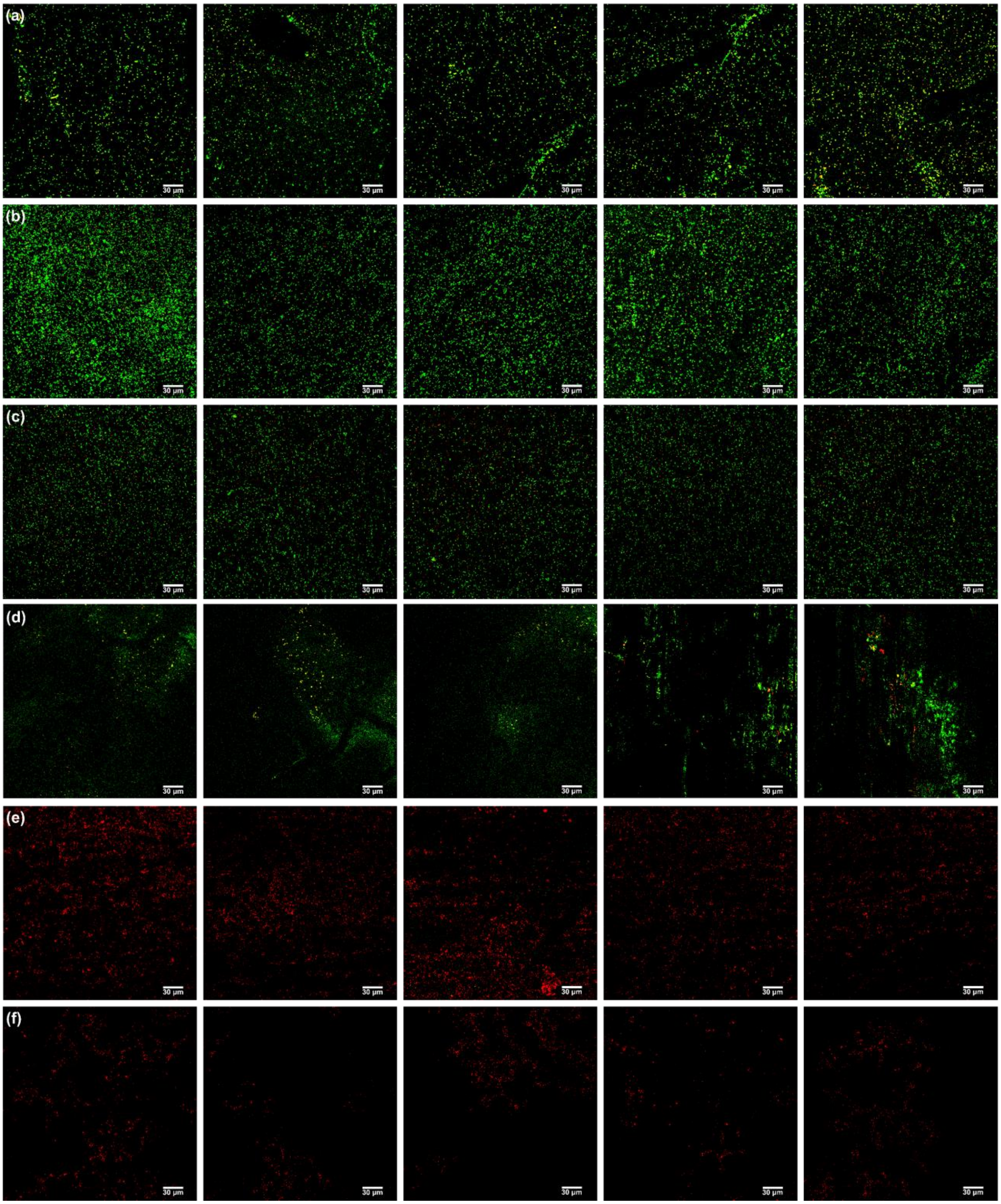

Figure S26. Confocal microscopy images of *S. aureus* after 72 hours of incubation on (a) control, (b) glass, (c) pure PDMS, (d) bare zinc, (e) ZnO nanopillars, (f) PDMS-coated nanopillars.

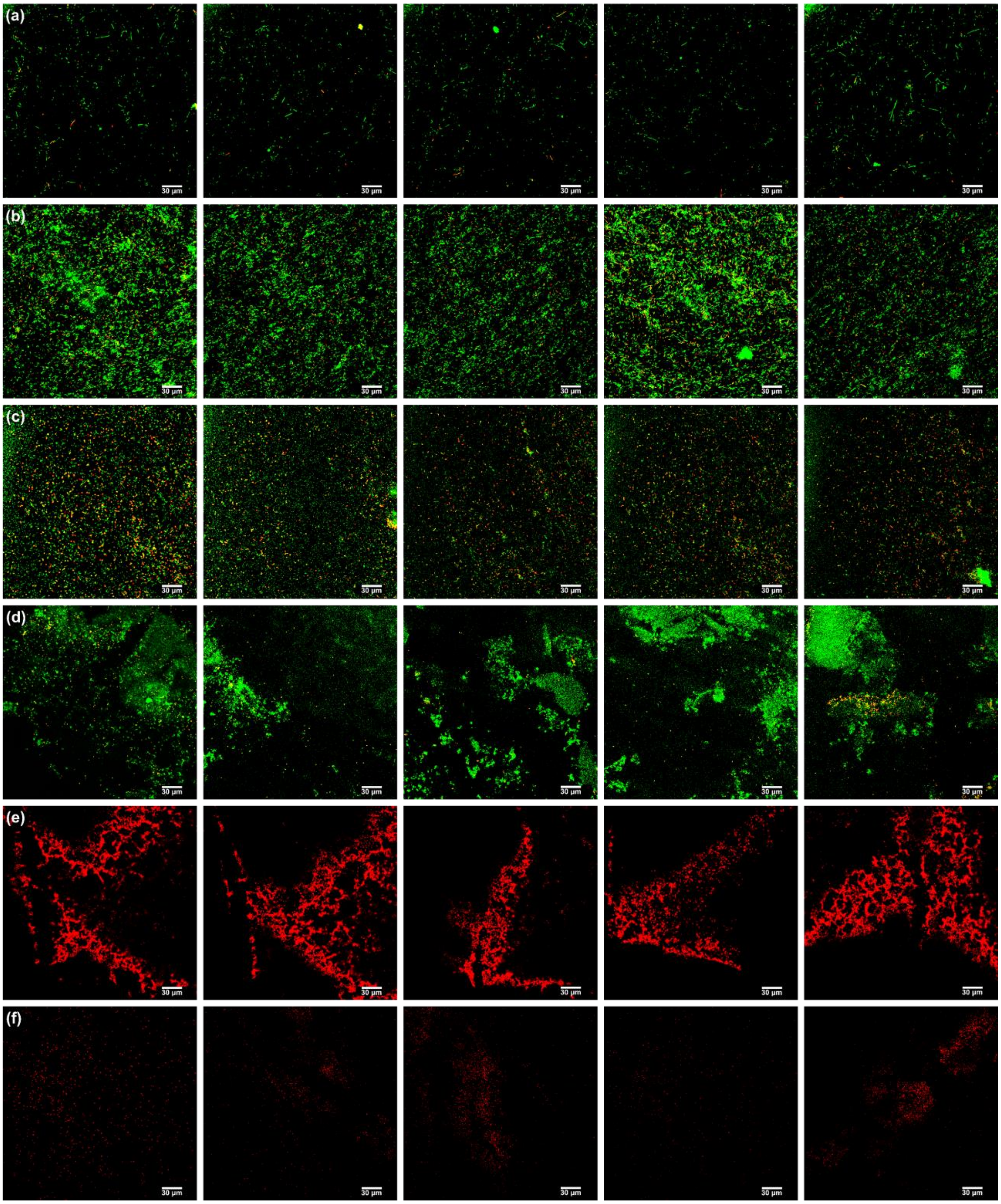

Figure S27. Confocal microscopy images of *E. coli* after 72 hours of incubation on (a) control, (b) glass, (c) pure PDMS, (d) bare zinc, (e) ZnO nanopillars, (f) PDMS-coated nanopillars.
